# *Mycobacterium tuberculosis* inhibits METTL14-mediated m^6^A methylation of *Nox2* mRNA and suppresses anti-TB immunity

**DOI:** 10.1038/s41421-024-00653-4

**Published:** 2024-03-29

**Authors:** Mingtong Ma, Yongjia Duan, Cheng Peng, You Wu, Xinning Zhang, Boran Chang, Fei Wang, Hua Yang, Ruijuan Zheng, Hongyu Cheng, Yuanna Cheng, Yifan He, Jingping Huang, Jinming Lei, Hanyu Ma, Liru Li, Jie Wang, Xiaochen Huang, Fen Tang, Jun Liu, Jinsong Li, Ruoyan Ying, Peng Wang, Wei Sha, Yawei Gao, Lin Wang, Baoxue Ge

**Affiliations:** 1 Shanghai Key Laboratory of Tuberculosis, Shanghai Pulmonary Hospital, Key Laboratory of Pathogen–Host Interaction, Ministry of Education, Tongji University School of Medicine, Shanghai, China; 2 Department of Microbiology and Immunology, Tongji University School of Medicine, Shanghai, China; 3 Institute for Regenerative Medicine, Shanghai East Hospital, Shanghai Key Laboratory of Signaling and Disease Research, School of Life Sciences and Technology, Tongji University, Shanghai, China; 4 State Key Laboratory of Protein and Plant Gene Research, Peking-Tsinghua Center for Life Sciences, School of Life Sciences, Peking University, Beijing, China; 5 State Key Laboratory of Cell Biology, Shanghai Key Laboratory of Molecular Andrology, CAS Center for Excellence in Molecular Cell Science, Shanghai Institute of Biochemistry and Cell Biology, Chinese Academy of Sciences, University of Chinese Academy of Sciences, Shanghai, China; 6 Clinic and Research Center of Tuberculosis, Shanghai Pulmonary Hospital, Tongji University School of Medicine, Shanghai, China; 7 Clinical Translation Research Center, Shanghai Pulmonary Hospital, Tongji University School of Medicine, Shanghai, China

**Keywords:** Pattern recognition receptors, Transcriptional regulatory elements

## Abstract

Internal N^6^-methyladenosine (m^6^A) modifications are among the most abundant modifications of messenger RNA, playing a critical role in diverse biological and pathological processes. However, the functional role and regulatory mechanism of m^6^A modifications in the immune response to *Mycobacterium tuberculosis* infection remains unknown. Here, we report that methyltransferase-like 14 (METTL14)-dependent m^6^A methylation of NAPDH oxidase 2 (*Nox2*) mRNA was crucial for the host immune defense against *M. tuberculosis* infection and that *M. tuberculosis*-secreted antigen EsxB (Rv3874) inhibited METTL14-dependent m^6^A methylation of *Nox2* mRNA. Mechanistically, EsxB interacted with p38 MAP kinase and disrupted the association of TAB1 with p38, thus inhibiting the TAB1-mediated autophosphorylation of p38. Interaction of EsxB with p38 also impeded the binding of p38 with METTL14, thereby inhibiting the p38-mediated phosphorylation of METTL14 at Thr72. Inhibition of p38 by EsxB restrained liquid–liquid phase separation (LLPS) of METTL14 and its subsequent interaction with METTL3, preventing the m^6^A modification of *Nox2* mRNA and its association with the m^6^A-binding protein IGF2BP1 to destabilize *Nox2* mRNA, reduce ROS levels, and increase intracellular survival of *M. tuberculosis*. Moreover, deletion or mutation of the phosphorylation site on METTL14 impaired the inhibition of ROS level by EsxB and increased bacterial burden or histological damage in the lungs during infection in mice. These findings identify a previously unknown mechanism that *M. tuberculosis* employs to suppress host immunity, providing insights that may empower the development of effective immunomodulators that target *M. tuberculosis*.

## Introduction

*M. tuberculosis* is an extremely successful intracellular pathogen that causes tuberculosis (TB), which is associated with 10 million active cases and 1.5 million deaths annually^1^. Upon *M. tuberculosis* infection, host cells launch a range of cellular innate immune responses; however, *M. tuberculosis* modulates innate defense mechanisms to promote its intracellular survival^2–7^.

One striking characteristic of *M. tuberculosis* is its utilization of different type VII secretion systems to secrete numerous proteins across their hydrophobic and impermeable cell walls into the cytoplasm of host macrophages. Several secreted proteins from *M. tuberculosis* have been shown to either stimulate or inhibit host innate immune responses^5^. EsxB, which is also named CFP10, is an *M. tuberculosis*-specific secretory protein encoded by an RD-1 region that is deleted from the vaccine strain *Mycobacterium bovis* bacille Calmette-Guérin (BCG)^8–10^. It is conventionally considered an *M. tuberculosis*-specific antigen for diagnosing TB and anti-TB vaccine design because of its ability to stimulate the production of IFN-γ by T lymphocytes^11,12^. EsxB-induced IFN-γ, TNF-α, and IL-10 are associated with clinical TB^13^. It has been shown that preincubation of macrophages with recombinant EsxB protein reduces the production of reactive oxygen and nitrogen species^8,14,15^. However, the exact role and mechanism of EsxB in the pathogenesis of *M. tuberculosis* infection remain largely unclear.

One important regulatory mechanism of gene expression is the chemical modification of RNA at the post-transcriptional level. *N*^6^-methyladenosine (m^6^A) is the most abundant modification in messenger RNA (mRNA) and non-coding RNA in eukaryotic cells^16–19^. The m^6^A modification of RNA is achieved by the writer complex, composed of such factors as methyltransferase-like 3 (METTL3), METTL14, Wilms’ tumor 1-associated protein (WTAP), and KIAA1429 and removed by the erasers fat mass and obesity-associated protein (FTO) and alkylated DNA repair protein ALKB homolog 5 (ALKBH5)^18^. The post-transcriptional m^6^A methylation of adenosines in RNA controls mRNA stability, nuclear processing, translation, RNA–protein interactions, and gene transcription^18,20–23^. Increasing evidence has revealed that the m^6^A methylation of RNA regulates many physiological processes, including development^18,19^, stem cell differentiation^24^, DNA damage repair^25^, and the circadian clock^26^. Moreover, dysfunctional m^6^A modification of RNA is associated with various diseases, including tumorigenesis^27^. However, whether and how m^6^A mRNA methylation is involved in the pathogenesis of *M. tuberculosis* remains unclear.

Accumulating evidence indicates that liquid–liquid phase separation (LLPS) is a vital and ubiquitous phenomenon whereby multivalent weak macromolecular interactions drive the transition of some proteins into the formation of biomolecular condensates or droplets that exhibit liquid characteristics^28–30^. These biomolecular condensates or droplets are micron-scale cellular compartments that lack membranous enclosures but selectively enhance protein density, thereby allowing higher rates of biochemical reactions. Recent studies indicate that LLPS plays a vital role in human health and disease^31^ and that dysregulation of LLPS leads to aberrant condensate and amyloid formation that can contribute to many human diseases, including neurodegeneration and cancer^32^. However, the role of LLPS in the regulation of immune responses to *M. tuberculosis* infection remains unclear.

Nicotinamide adenine dinucleotide phosphate (NADPH) oxidases (NOX) are enzymes that catalyze the reduction of molecular oxygen to generate superoxide (O_2_^−^) and hydrogen peroxide (H_2_O_2_) by utilizing NADPH as an electron donor^33^. Superoxide is converted to H_2_O_2_, which is further converted to additional reactive oxygen species (ROS)^34,35^. There are seven enzymes in the NOX family: NOX1-5 and dual oxidase (DUOX) 1–2. NOX enzymes in humans play important roles in diverse biological functions and vary in expression from tissue to tissue. Importantly, NOX2 is involved in regulating many aspects of innate and adaptive immunity, including regulation of type I interferons, the inflammasome, phagocytosis, antigen processing and presentation, and cell signaling^36^. Targeting NOX enzymes directly or through scavenging free radicals has been proven to be therapeutically useful for treating autoimmunity and acute lung injury where oxidative stress contributes to pathology^36^. ROS are produced by NOX2 upon the phagocytosis of pathogens in macrophages to facilitate the killing of *M. tuberculosis*^37^. However, whether and how *Nox2* is regulated by m^6^A mRNA methylation has not been reported.

In the present study, we uncover a critical role of m^6^A mRNA methylation of *Nox2* by METTL14 in regulating the anti-mycobacterial immune response, whereby *M. tuberculosis*-secreted antigen EsxB suppresses the m^6^A RNA modification of *Nox2* to promote its intracellular survival. We provide molecular and genetic evidence demonstrating that EsxB inhibits p38-mediated phosphorylation as well as LLPS of METTL14, thereby reducing the m^6^A methylation of *Nox2* mRNA and the production of ROS and promoting the intracellular survival of *M. tuberculosis*.

## Results

### METTL14 regulates the intracellular survival of *M. tuberculosis*

**Fig. 1:**
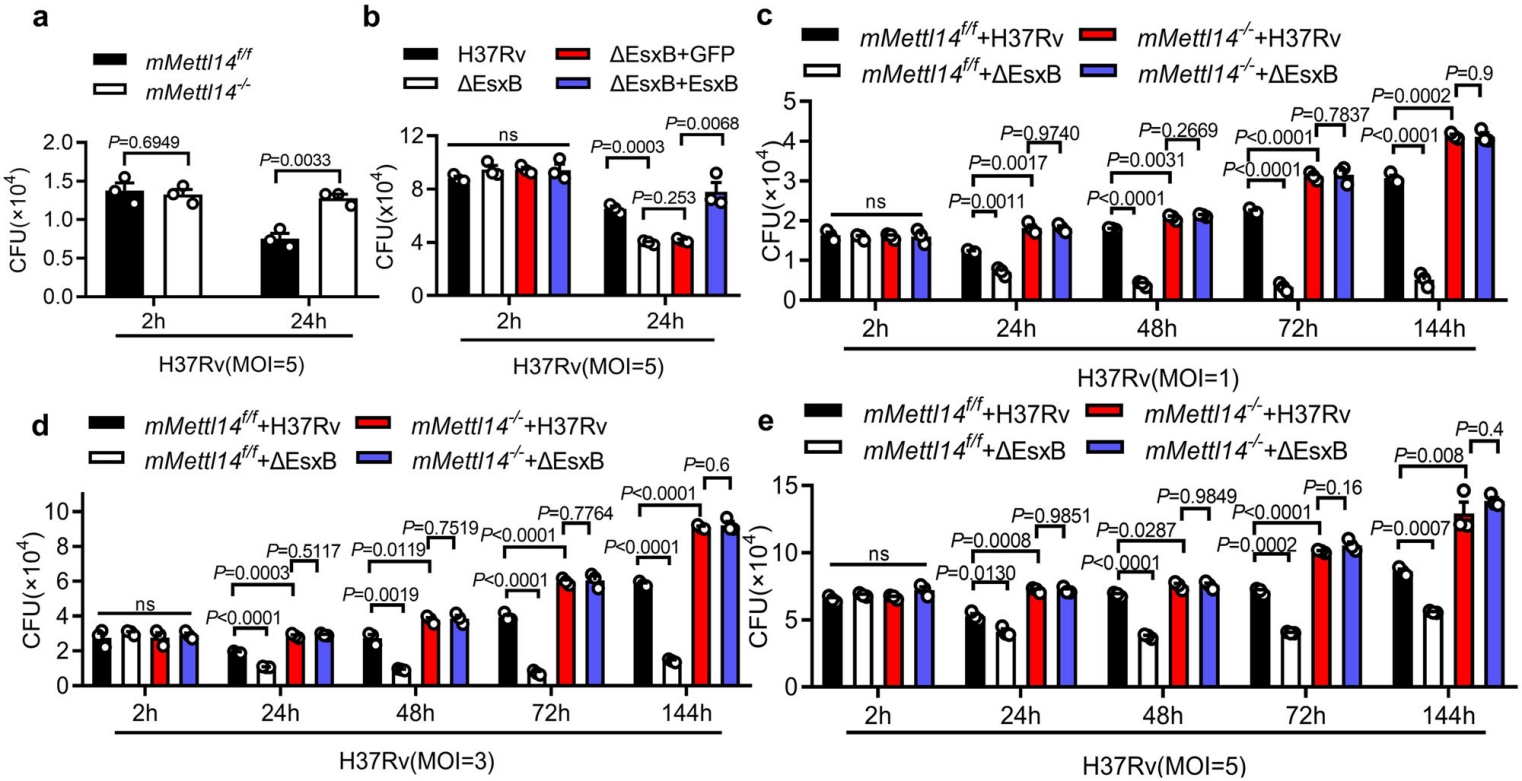
EsxB promotes survival of *M. tuberculosis* via METTL14. **a** CFU assay in peritoneal macrophages from control (*mMettl14*^*f/f*^) or *Mettl14* conditional knockout (*mMettl14*^*−/−*^) mice infected with H37Rv for 2 h and 24 h (MOI = 5). **b** Mouse peritoneal macrophages were infected with H37Rv, H37Rv:ΔEsxB, H37Rv(ΔEsxB+GFP), H37Rv(ΔEsxB+EsxB) for 2 h and 24 h, and then subjected to CFU assay (MOI = 5). **c–e** *mMettl14*^*f/f*^ or *mMettl14*^*−/−*^ mouse peritoneal macrophages infected with H37Rv, H37RvΔEsxB for 2, 24, 48, and 144 h, and then subjected to CFU assay (MOI = 1, 3, 5). Data in **a**–**e** reflect mean ± SEM from three independent biological replicates; two-tailed unpaired Student’s *t*-test (**a**–**e**) was used for statistical analysis.

To investigate whether m^6^A mRNA methylation is involved in innate immune responses to *M. tuberculosis*, we transiently transfected mouse peritoneal macrophages with siRNA that targeted RNA methyltransferase METTL14 and analyzed the effects on the intracellular survival of *M. tuberculosis* in a colony-forming unit (CFU) assay. Silencing of *Mettl14*^38^ significantly promoted the ~97% intracellular survival *M. tuberculosis* in macrophages compared to the control (Supplementary Fig. S1a, b). We next crossed *Mettl14*^*flox/flox*^ (*mMettl14*^*f/f*^) mice with *LysM-Cre* transgenic mice and generated *Mettl14*^*flox/flox*^;*LysM-Cre* (*mMettl14*^*−/−*^) mice that carried *Mettl14* gene deletion in myeloid cells. Using western blotting assay, we confirmed *Mettl14* deletion in peritoneal macrophages from *mMettl14*^*−/−*^ mice (Supplementary Fig. S1c). Consistently, knockout of *Mettl14* in macrophages also increased the intracellular bacteria CFU count at 24 h post-infection (Fig. 1a), while no significant difference in CFU count was observed between *mMettl14*^*f/f*^ and *mMettl14*^*−/−*^ at 2 h post-infection suggesting that the macrophages isolated from *mMettl14*^*−/−*^ mice may have a deficiency in limiting intracellular *M. tuberculosis*. *Mettl14* deficiency had no significant effect on the cell death, indicated by lactate dehydrogenase (LDH) release (Supplementary Fig. S1d), as well as macrophage polarization, as indicated by M1 markers CD86 and MHCII and the M2 marker CD206 (Supplementary Fig. S1e). When infected with *M. tuberculosis* H37Rv, an equivalent expression of *Il1b* and *Il6* was detected in *mMettl14*^*f/f*^ and *mMettl14*^*−/−*^ at 4 h post-infection (Supplementary Fig. S1f, g), indicating that Mettl14 may not regulate macrophages inflammatory response. These results suggest that METTL14 may regulate the intracellular survival of *M. tuberculosis*.

METTL14 and METLL3 form a heterodimer in which METTL3 is the catalytic component, and METTL14 facilitates the binding of METTL3 to the RNA substrate^39^. We also used the METTL3-specific inhibitor STM2457^40^ to examine whether the function of METTL14 is m^6^A-dependent. Inhibition of METTL3 by STM2457 enhanced the survival of *M. tuberculosis* in *mMettl14*^*f/f*^ macrophages to a level similar to that in *mMettl14*^−/−^ (Supplementary Fig. S1h). Finally, complementation of *mMettl14*^−/−^ macrophages with wild-type (WT) Mettl14, but not with Mettl14 (R298E), a mutant defective in the binding with METTL3^41^, rescued the intracellular survival of *M. tuberculosis* (Supplementary Fig. S1h). Altogether, these results suggest that METTL14 is a novel host factor that restricts the intracellular survival of *M. tuberculosis* through its methyltransferase-related function.

### EsxB promotes the intracellular survival of *M. tuberculosis* via METTL14

To evaluate whether *M. tuberculosis*-secreted proteins modulate host m^6^A RNA modification to achieve immune evasion, we infected primary peritoneal macrophages with several *M. tuberculosis*-secreted protein mutant strains individually, including H37Rv:∆EsxB^10^, H37Rv:ΔureC^42^, and H37Rv:ΔnuoG^43^ and performed a CFU assay. Although the intracellular survival of *M. tuberculosis* markedly decreased in control macrophages that were infected with all these mutant strains (Fig. 1b; Supplementary Fig. S1i, j), only the lower survival of H37Rv:ΔEsxB was restored in macrophages whose *Mettl14* gene was knocked down or knock out (Fig. 1c–e; Supplementary Fig. S1i, k) at 24 h post infection. We further performed CFU assay to examine the intracellular growth of H37Rv and H37Rv:ΔEsxB (MOI = 1, 3, 5) at 2, 24, 48, 72, and 144 h post infection. Under all experimental conditions in Mettl14-intact macrophages, the intracellular survival of the EsxB deletion mutant was significantly lower than that of the WT H37Rv strain, especially up to 6 days post-infection. However, the deletion of *Mettl14* eliminated EsxB-mediated intracellular CFU increase (Fig. 1c–e). All these data suggested that EsxB may promote the intracellular survival of *M. tuberculosis* through METTL14.

### EsxB inhibits the stability of *Nox2* mRNA

**Fig. 2:**
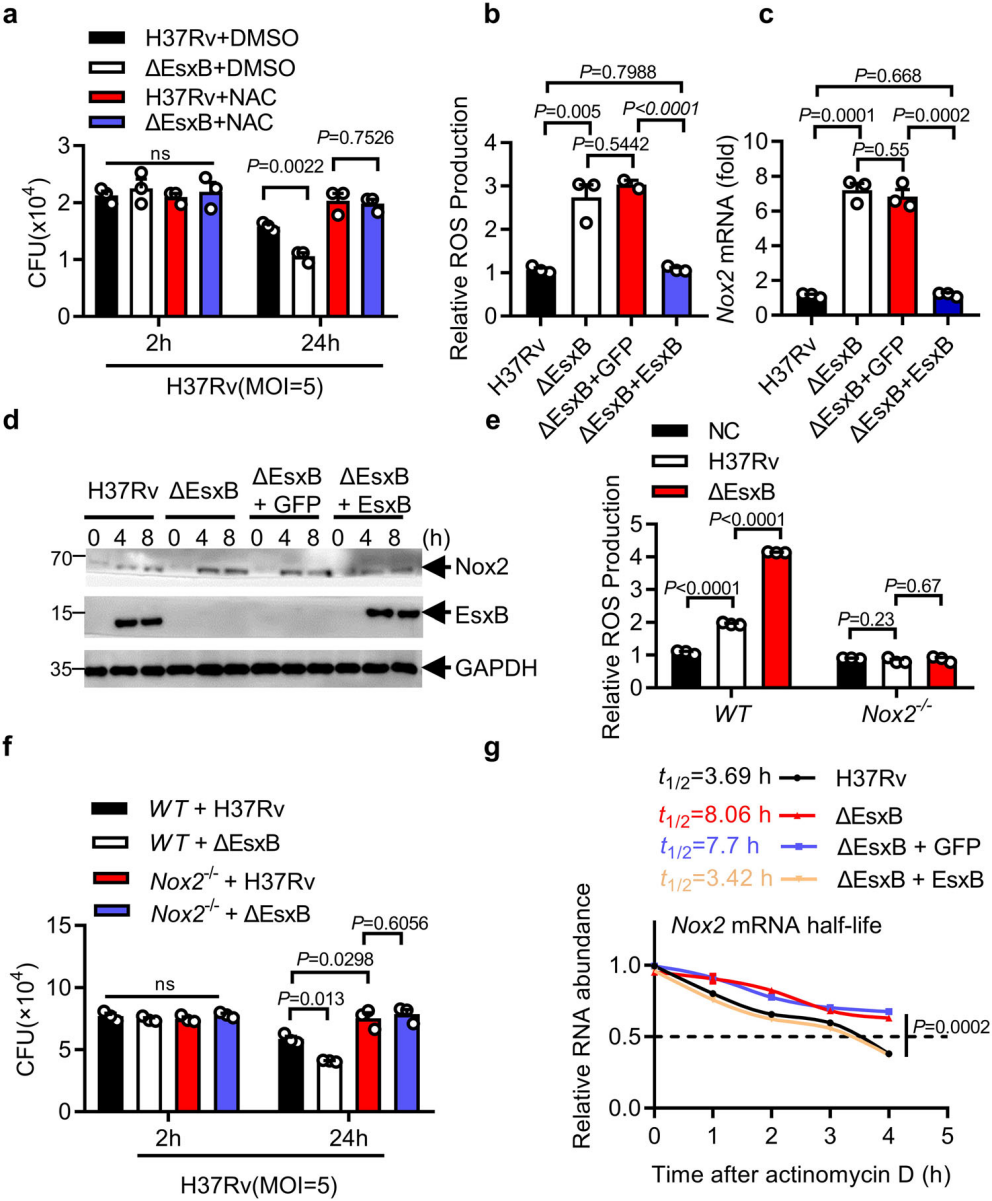
EsxB inhibits NOX2. **a**, **b** Mouse peritoneal macrophages pre-treated with DMSO, NAC (ROS inhibitor) and infected with H37Rv or H37Rv(ΔEsxB) (MOI = 5) for 2 and 24 h, and then subjected to CFU assay (MOI = 5). **b** Changes in the levels of ROS (DCF staining; green) in the macrophages upon H37Rv, H37Rv:ΔEsxB, H37Rv(ΔEsxB+GFP) and H37Rv(ΔEsxB+EsxB) infection for 4 h (MOI = 5). **c**, **d** qPCR analysis of *Nox2* mRNA level (**c**) and immunoblot (IB) of Nox2 protein levels (**d**) from peritoneal macrophages infected with H37Rv, H37Rv:ΔEsxB, H37Rv(ΔEsxB+GFP) and H37Rv(ΔEsxB+EsxB) (MOI = 5) for 4 h. **e** Peritoneal macrophages from WT or *Nox2*^*−/−*^ mice were infected with H37Rv, H37Rv(ΔEsxB) for 4 h, and then the ROS level was detected (MOI = 5). **f** CFU assay in peritoneal macrophages from WT or *Nox2*^*−/−*^ mice infected with H37Rv or H37Rv(ΔEsxB) for 2 h and 24 h, and then subjected to CFU assay (MOI = 5). **g** *Nox2* mRNA stability analysis in macrophages after actinomycin D treatment. Mouse peritoneal macrophages were infected with H37Rv, H37Rv:ΔEsxB, H37Rv(ΔEsxB + GFP), and H37Rv(ΔEsxB + EsxB) (MOI = 5) for 4 h and treated with actinomycin D (5 μg/mL). Cells were harvested at the indicated timepoints. Expression levels were normalized to 0 h and *Gapdh* was used as a reference gene. Results in **d** are representative images from one of three independent experiments. All of the results (except **d**) reflect the mean ± SEM from three independent biological experiments. Two-tailed unpaired Student’s *t*-test were used in **a**–**c** and **e**–**g**.

ROS production, apoptosis, autophagy, and lysosomal acidification are crucial cellular antimicrobial events that limit the intracellular survival of mycobacteria^44^. Treatment with the ROS inhibitor N-acetylcysteine (NAC)^45^ provided nearly complete abrogation of the enhanced effect of EsxB on the survival of *M. tuberculosis* in macrophages (Fig. 2a). We used a 2′,7′-dichlorofluorescein-diacetate (DCFH-DA) fluorescent probe to detect ROS levels^34^ and found that macrophages that were infected with H37Rv:ΔEsxB exhibited much higher ROS levels compared with macrophages that were infected with WT H37Rv (Fig. 2b). Complementation of H37Rv:ΔEsxB with WT EsxB, but not with GFP, inhibited ROS production (Fig. 2b). It has been shown that deletion of EsxB also abolishes the secretion of EsxA (ESAT-6)^46,47^. Consistently, our data demonstrated that neither EsxB nor EsxA was observed in culture filtrates of H37Rv:ΔEsxB (Supplementary Fig. S2a). It has been shown that EsxA promotes ROS production^48–50^, thus the observed effect of H37Rv:ΔEsxB on ROS production is unlikely to be due to EsxA deficiency. To examine this, we expressed EsxA in immortalized bone marrow-derived macrophages (iBMDM) cells and infected them with H37Rv or H37Rv:ΔEsxB. Expression of EsxA in H37Rv:ΔEsxB-infected macrophages did not significantly change the enhanced effect of ΔEsxB on ROS production (Supplementary Fig. S2b). Together, these results suggest that the observed effects of H37Rv:ΔEsxB on ROS production may primarily be mediated through EsxB deficiency but not EsxA deficiency. It is established that type II interferons play a mandatory role in limiting the survival of *M. tuberculosis*^51^. Thus, we analyzed the expression level of *Ifng* in *mMettl14*^*f/f*^ and *mMettl14*^*−/−*^ macrophage infected with H37Rv, H37Rv:ΔEsxB, and H37Rv:ΔEsxB+EsxB strains. As shown in Supplementary Fig. S2c, the deletion of EsxB had no significant effects on interferon expression. Thus, EsxB may promote the intracellular survival of *M. tuberculosis* by inhibiting ROS production. To further investigate the underlying mechanism, we conducted overall pathway analysis in mouse peritoneal macrophages infected with *M. tuberculosis* H37Rv or H37Rv:∆EsxB by using RNA sequencing (RNA-seq) and subsequent validation via quantitative real-time PCR (qPCR). As shown in Fig. 2c and Supplementary Fig. S2d, e, *Nox2* is one of the most significantly inhibited genes by EsxB in H37Rv-infected macrophages. Consistently with this, macrophages infected with H37Rv:ΔEsxB had much higher Nox2 protein levels than macrophages infected with H37Rv, suggesting that EsxB may inhibit *Nox2* mRNA levels (Fig. 2d). In addition, tumor necrosis factor gene (*Tnf*)^52,53^, chemokine and chemotactic receptor-related genes (*C5ar1*, *Ccl4*, and *Mcoln2*), formyl peptide receptor gene (*Fpr1*), insulin-like growth factor (*Igf1*), adhesion molecule (*Clmp*), and the interferon-gamma inducible gene (*Ifi47*) were also found to be among the top ten genes that were most significantly inhibited by EsxB, suggesting an inhibitory effect of EsxB on the expression of these pathways.

To examine whether EsxB suppresses ROS production via *Nox2*, we detected ROS production in WT or *Nox2*^*−/−*^ peritoneal macrophages infected with H37Rv and H37Rv:ΔEsxB (Supplementary Fig. S2f). As shown in Fig. 2e, deletion of *Nox2* nearly eliminated EsxB-mediated inhibition of ROS production. The knockdown or knockout of *Nox2* markedly attenuated the enhanced effect of EsxB on the survival of *M. tuberculosis* in macrophages (Fig. 2f; Supplementary Fig. S2g, h) but had no significant effect on *M. tuberculosis*-induced cell death (Supplementary Fig. S2i). Altogether, these results suggest that EsxB may suppress ROS production by inhibiting *Nox2* mRNA levels, thereby promoting the intracellular survival of *M. tuberculosis*.

Given that EsxB inhibits *Nox2* mRNA levels in *M. tuberculosis*-infected macrophages, which may result from post-transcriptional effects of mRNA stability, we treated WT H37Rv- and H37Rv:ΔEsxB-infected primary peritoneal macrophages with the transcription inhibitor actinomycin D^54^, examined *Nox2* mRNA levels, and calculated the rate of the half-life of *Nox2* mRNA. Both the level and half-life of *Nox2* mRNA were substantially higher in H37Rv:ΔEsxB-infected macrophages than in macrophages infected with WT H37Rv (Fig. 2c, g), suggesting that EsxB may inhibit the stability of *Nox2* mRNA. To examine whether EsxB specifically inhibits the stability of *Nox2* mRNA, we treated H37Rv- or H37Rv:ΔEsxB-infected peritoneal macrophages with actinomycin D, and then analyzed the mRNA levels of key regulators of the ROS- and RNS-related genes, including *Nox1-5*, *Nrf2*, *Duox1*, *Xor*, *Sod1*, *iNos* and *Pink1*. The data showed that only *Nox2* mRNA stability was significantly inhibited by EsxB (Supplementary Fig. S2j). These results suggest that EsxB may specifically inhibit the stability of *Nox2* mRNA in *M. tuberculosis-*infected macrophages.

### METTL14 regulates the m^6^A methylation of *Nox2* mRNA

**Fig. 3:**
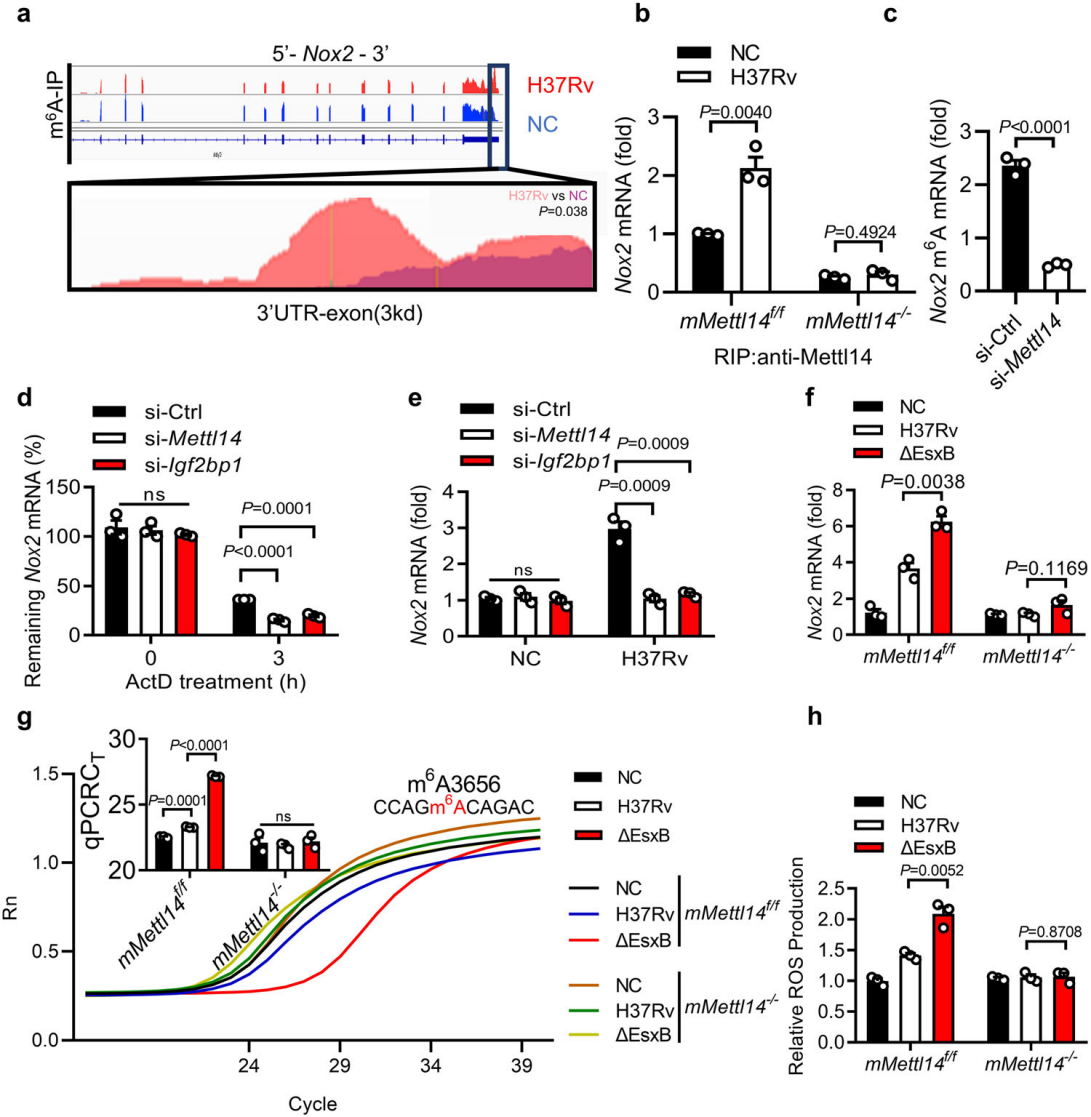
EsxB inhibits m^6^A methylation of *Nox2* mRNA via Mettl14. **a** MeRIP-seq data of *Nox2* in macrophages infected with H37Rv for 2 h. **b** RIP-qPCR for *Nox2* mRNA in *mMettl14*^*f/f*^ and *mMettl14*^*−/−*^ peritoneal macrophages infected with H37Rv for 2 h (MOI = 5) by using anti-Mettl14 antibody. Rabbit IgG served as control. **c** MeRIP-qPCR analysis of relative m^6^A *Nox2* mRNA in si-*Ctrl* or si-*Mettl14* macrophages infected with H37Rv for 2 h. **d** *Nox2* mRNA stability analysis in si-*Ctrl*, si-*Mettl14* or *Igf2bp1*-knockdown (si-*Igf2bp1*) mouse peritoneal macrophages infected with H37Rv for 3 h (MOI = 5) and treated with 5 μg/mL actinomycin D (ActD) for another 4 h. **e** *Nox2* mRNA levels analysis in si-*Ctrl*, si-*Mettl14* or si-*Igf2bp1* mouse peritoneal macrophages infected with H37Rv for 3 h (MOI = 5). **f** qPCR analysis of *Nox2* mRNA from *mMettl14*^*f/f*^ and *mMettl14*^*−/−*^ mouse macrophages infected with H37Rv or H37Rv(ΔEsxB) (MOI = 5) for 4 h. **g** Real-time fluorescence amplification curves and bar plot of the threshold cycle (*C*_T_) of qPCR showing SELECT results for detecting m^6^A3656 in *Nox2* mRNA in *mMettl14*^*f/f*^ and *mMettl14*^*−/−*^ mouse peritoneal macrophages infected with H37Rv and H37Rv:ΔEsxB for 2 h (MOI = 5). Rn is the raw fluorescence for the associated well normalized to the fluorescence of the passive reference dye (ROX). **h** Changes in the levels of ROS (DCF staining; green) in the *mMettl14*^*f/f*^ or *mMettl14*^*−/−*^ macrophages infected with H37Rv or H37Rv(ΔEsxB) for 4 h (MOI = 5). Results in **b**–**h** reflect the mean ± SEM from three independent biological experiments. Two-tailed unpaired Student’s *t*-test were used in (**b**–**h**).

Considering the major effect of m^6^A RNA methylation on mRNA lifespan^55^, we sought to determine whether *Nox2* RNA is modified by m^6^A methylation in *M. tuberculosis*-infected macrophages. To examine the abundance of m^6^A methylation on *Nox2* transcripts, total RNA from uninfected and H37Rv-infected macrophages was subjected to m^6^A immunoprecipitation and methylated RNA immunoprecipitation sequencing (MeRIP)^56^. By scoring 24,029 and 24,723 m^6^A peaks in uninfected and H37Rv-infected macrophages, respectively, higher m^6^A enrichment on *Nox2* RNA was found (Fig. 3a). m^6^A RNA methylation mostly occurs on conserved RRACH sequence motifs, in which R denotes A or G, and H denotes A, C, or U^57^. We found several RRACH motifs on the *Nox2* sequences (Supplementary Fig. S3a). Using RNA immunoprecipitation (RIP)^58^, we found that METTL14 bound to *Nox2* mRNA in *M. tuberculosis*-infected primary macrophages (Fig. 3b). We also detected Mettl14 in the pulldown of m6A-modified mRNAs in H37Rv infected peritoneal macrophages (Supplementary Fig. S3b). Moreover, *Mettl14* knockdown in primary peritoneal macrophages significantly reduced m^6^A modification levels of *Nox2* mRNA in response to *M. tuberculosis* infection (Fig. 3c). To examine whether methylation by METTL14 is specific to *Nox2* mRNA, we analyzed the mRNA methylation level of several ROS-and RNS-related genes including *Nox1-5*, *Nrf2*, *Duox1*, *Xor*, *Sod1*, *iNos* and *Pink1* from H37Rv:ΔEsxB-infected *mMettl14*^*f/f*^ or *mMettl14*^*−/−*^ peritoneal macrophages. As shown in Supplementary Fig. S3c, methylation by METTL14 was particularly extensive for the mRNA of the *Nox2* gene, but not for the other genes. These results suggest that METTL14 may specifically mediate the methylation of *Nox2* mRNA. To our knowledge, these data demonstrate for the first time that METTL14 modulates the m^6^A methylation of *Nox2* mRNA during *M. tuberculosis* infection.

It is reported that insulin-like growth factor 2 mRNA-binding proteins (IGF2BPs) impair mRNA decay by interfering with mRNA targeted by endonucleases^59^. The silencing of either *Mettl14* or *Igf2bp1* by siRNA or deletion of *Mettl14* resulted in a decrease in the stability of *Nox2* mRNA in *M. tuberculosis*-infected primary macrophages (Fig. 3d; Supplementary Fig. S3d, e). The binding of *Nox2* mRNA with IGF2BP1 was also validated by RNA immunoprecipitation sequencing (RIP-seq) and RNA immunoprecipitation quantitative real-time PCR (RIP-qPCR), suggesting that Nox2 is a target mRNA of IGF2BP1 (Supplementary Fig. S3f, g). Consistent with this finding, *Mettl14-* and *Igf2bp1-*knockdown by siRNA or deletion *Mettl14* led to much lower levels of *Nox2* mRNA in macrophages infected with H37Rv *M. tuberculosis* (Fig. 3e, f; Supplementary Fig. S3h). These results suggest that the m^6^A methylation pathway may regulate *Nox2* mRNAs in a post-transcriptional and IGF2BP1-dependent manner.

IGF2BP1/2/3 are a new family of m^6^A readers that guard m^6^A-modified mRNAs from decay^60–62^. To further explore the potential redundant functions of other IGF2BPs in regulating *Nox2* mRNA stability in H37Rv-infected macrophages, we detected the *Igf2bp1/2/3* expression and found that *Igf2bp1* had the most abundant mRNA level in *M. tuberculosis* H37Rv-infected macrophages (Supplementary Fig. S3i). Moreover, silencing of *Igf2bp2* or *Igf2bp3* in macrophages had no significant effect on EsxB-mediated reduction of mRNA stability, mRNA content, or protein level of *Nox2* (Supplementary Fig. S3j–l). Together, these results suggested that IGF2BP1, not other IGF2BP proteins, may modulate the mRNA stability of *Nox2* in *M. tuberculosis* H37Rv-infected macrophages.

### EsxB inhibits the m^6^A methylation of *Nox2* mRNA via METTL14

We next investigated whether EsxB inhibits the m^6^A methylation of *Nox2* mRNA via METTL14. By using MeRIP-qPCR, we found the knockdown of *Mettl14* by specific siRNA or deletion of *Mettl14* reduced the m^6^A methylation of *Nox2* mRNA in H37Rv:ΔEsxB-infected macrophages to levels comparable to cells infected with H37Rv (Supplementary Fig. S3m, n). We also determined the methylation sites by performing single-base elongation- and ligation-based qPCR amplification (SELECT) analysis for the detection of single m^6^A locus at single-base resolution^63^, and found that m^6^A levels of A3656 in the 3′ UTR of *Nox2* were significantly increased upon H37Rv(ΔEsxB) infection, compared to those infected with H37Rv, but this increment was not observed in *mMettl14*^*−/−*^ macrophages (Fig. 3g). Deletion of *Mettl14* in macrophages abolished the EsxB-mediated *Nox2* decrement (Supplementary Fig. S3o). Moreover, the knockout of *Mettl14* eliminated the inhibitory effect of EsxB on ROS production in H37Rv-infected macrophages (Fig. 3h). Although EsxB showed little inhibition on mitochondrial ROS, the deletion of *Mettl14* did not significantly change EsxB-mediated mitochondrial ROS production (Supplementary Fig. S3p). These results suggest that EsxB may inhibit the METTL14-mediated m^6^A methylation of *Nox2* mRNA, thereby reducing ROS production during *M. tuberculosis* infection.

### LLPS of METTL14 is dependent on Thr72

**Fig. 4:**
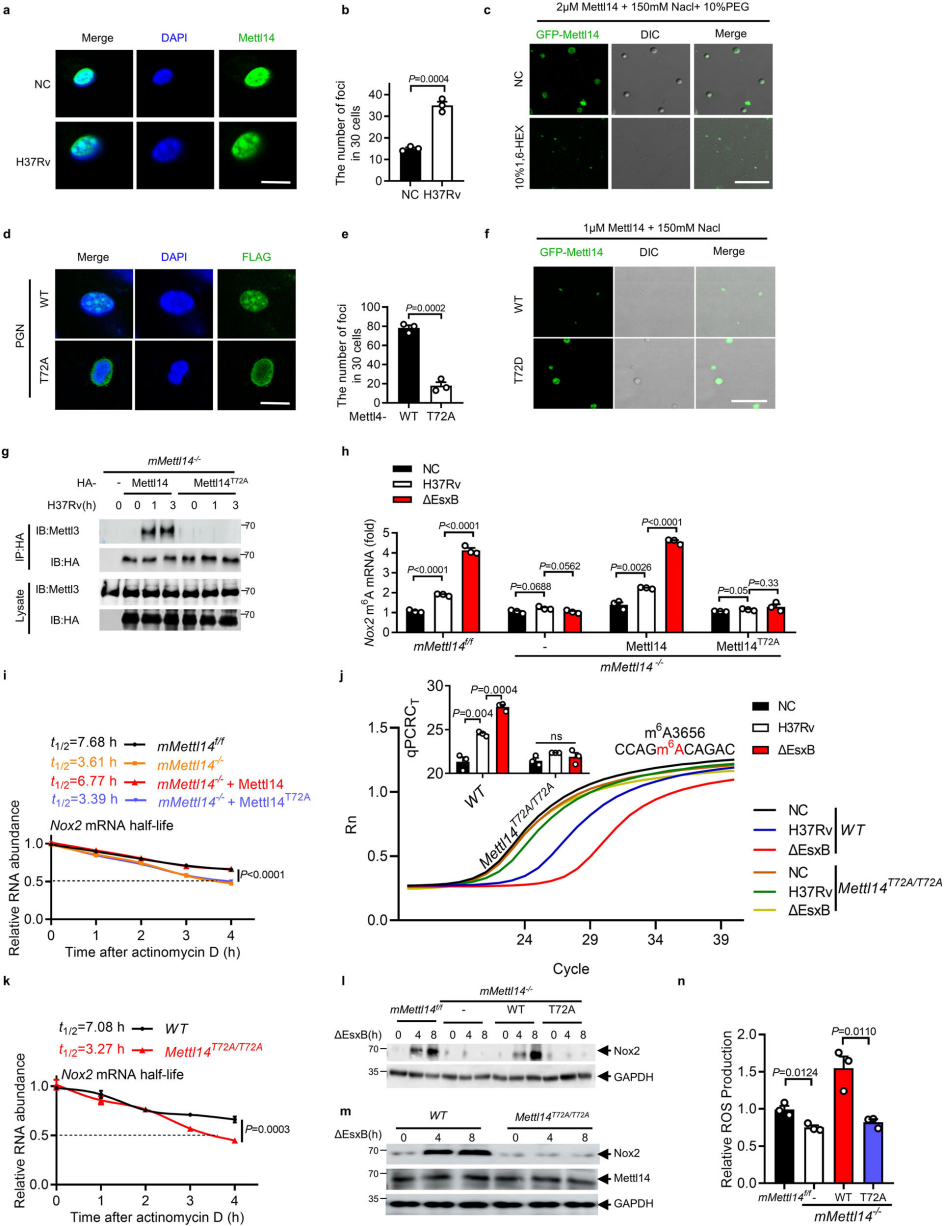
LLPS of METTL14 is dependent on Thr72. **a**, **b** Immunofluorescence staining for METTL14 in mouse peritoneal macrophages infected with H37Rv for 2 h (MOI = 5), Scale bar, 10 μm. Every point in **b** represents the number of Mettl14 foci in 30 cells. The foci in the nucleus with a diameter over 200 nm were included. **c** METTL14 forms liquid droplets in vitro. Scale bar, 5 μm. **d**, **e** Immunofluorescence staining of METTL14 in iBMDM cells expressing FLAG-Mettl14-WT or FLAG-Mettl14-T72A stimulated with PGN for 2 h. Scale bar, 10 μm. Every point represents the number of Mettl14 foci in 30 cells. The foci in the nucleus with a diameter over 200 nm were included. **f** LLPS of GFP-Mettl14-WT and GFP-Mettl14-T72D. **g** IB and immunoprecipitation (IP) in *mMettl14*^*−/−*^ macrophages transfected with plasmids encoding HA-tagged WT or T72A mutants of Mettl14. **h** MeRIP-qPCR analysis of relative m^6^A *Nox2* mRNA in *mMettl14*^*f/f*^ or *mMettl14*^*−/−*^ macrophages transfected with vector (-) or plasmids encoding FLAG tagged WT or T72A mutants of Mettl14 and infected with H37Rv or H37Rv(ΔEsxB) for 2 h (MOI = 5). Rabbit IgG served as control. **i** *Nox2* mRNA stability analysis in *mMettl14*^*f/f*^ or *mMettl14*^*−/−*^ macrophages transfected with vector or plasmids encoding FLAG-tagged WT or T72A mutants of Mettl14 after actinomycin D treatment. Macrophages were infected with H37Rv(ΔEsxB) for 4 h (MOI = 5) before being treated with actinomycin D (5 μg/mL). Real-time fluorescence amplification curves and bar plot of the threshold cycle (C_T_) of qPCR showing SELECT results for detecting m^6^A3656 in *Nox2* mRNA (**j**) and *Nox2* mRNA stability (**k**) in peritoneal macrophages from *WT* or *Mettl14*^*T72A*^ mice infected with H37Rv(ΔEsxB) for 2 or 4 h (MOI = 5). Rn is the raw fluorescence for the associated well normalized to the fluorescence of the passive reference dye (ROX). Changes in the Nox2 protein levels in *mMettl14*^*f/f*^ and *mMettl14*^*−/−*^ macrophages transfected with vector (-) or plasmids encoding FLAG-tagged WT or T72A mutants of Mettl14 (**l**), or in *WT* and *Mettl14*^*T72A*^ macrophages (**m**) infected with H37Rv(ΔEsxB) for 0, 4, 8 h (MOI = 5). **n** Changes in the levels of ROS (DCF staining; green) in *mMettl14*^*f/f*^ or *mMettl14*^*−/−*^ macrophages transfected with vector (-) or plasmids encoding FLAG-tagged WT or T72A mutants of Mettl14 infected with H37Rv for 4 h (MOI = 5). Results in **g**, **l** and **m** are representative images from one of three independent experiments. Results in **c** and **f** were repeated twice independently. Results in **b**, **e**, **h**–**k**, **n** reflect the mean ± SEM from three independent biological experiments. Two-tailed unpaired Student’s *t*-test were used in **b**, **e**, **h**, **j**, **n**.

In the nucleus of *M. tuberculosis-*infected macrophages, we frequently observed the robust induction of METTL14 condensates (Fig. 4a, b; Supplementary Fig. S4a). To examine whether METTL14 forms these condensates through LLPS in vitro, we purified recombinant GFP-METTL14 protein from *Escherichia coli* BL21. The addition of 2 μM METTL14 protein induced robust protein droplets that dramatically decreased with the addition of 10% 1,6-hexanediol (1,6-HEX; an inhibitor of LLPS) (Fig. 4c). When fluorescence recovery after photobleaching (FRAP) was performed 10 min after the initiation of phase separation, METTL14 fluorescence did not efficiently recover (Supplementary Fig. S4b, c), suggesting that METTL14 droplets are less dynamic in vitro.

To examine whether post-translational modifications of intrinsically disordered regions (IDRs) regulate METTL14 phase separation, we analyzed the sequence of the IDR in METTL14 and found that Thr72 is a conserved site across various species (Supplementary Fig. S4d). The substitution of threonine at 72 to alanine (T72A) abolished the peptidoglycan (a major component of the *M. tuberculosis* cell wall)-induced formation of METTL14 condensates in the nucleus of macrophages that overexpressed FLAG-METTL14 or FLAG-METTL14^T72A^ (Fig. 4d, e). Moreover, T72D mutant of METTL14, the phosphorylated form, formed more droplets in vitro (Fig. 4f). These data suggest that *M. tuberculosis* infection may induce the Thr72-dependent LLPS of METTL14 in macrophages.

We next investigated whether the LLPS of METTL14 regulates METTL14–METTL3 complex formation and, subsequently m^6^A RNA methylation. *M. tuberculosis* infection dramatically induced the formation of the METTL14–METTL3 complex in macrophages that were transfected with *Mettl14* but not the LLPS-defective T72A mutant of *Mettl14* (Fig. 4g). The transfection of *mMettl14*^*−/−*^ peritoneal macrophages with WT Mettl14 rather than the T72A mutant restored the m^6^A methylation and stability of *Nox2* mRNA (Fig. 4h, i). In *Mettl14*^*T72A/T72A*^ knock-in mice (Supplementary Fig. S4e), we found that *Mettl14*^*T72A/T72A*^ macrophages infected with *M. tuberculosis* H37Rv exhibited significantly less *Nox2* mRNA m^6^A methylation and reduced mRNA stability (Fig. 4j, k; Supplementary Fig. S4f). Consistently, we observed much lower Nox2 protein level in *Mettl14*^*T72A/T72A*^ knock-in macrophages or *mMettl14*^*−/−*^ macrophages complemented with Mettl14(T72A) (Fig. 4l, m). Moreover, the decrease in ROS production in *mMettl14*^*−/−*^ peritoneal macrophages recovered when complemented with WT *Mettl14* but not the T72A mutant (Fig. 4n). These results suggest that Thr72-dependent LLPS is essential for METTL14 to modulate the m^6^A methylation of *Nox2* mRNA.

### EsxB inhibits LLPS of METTL14

**Fig. 5:**
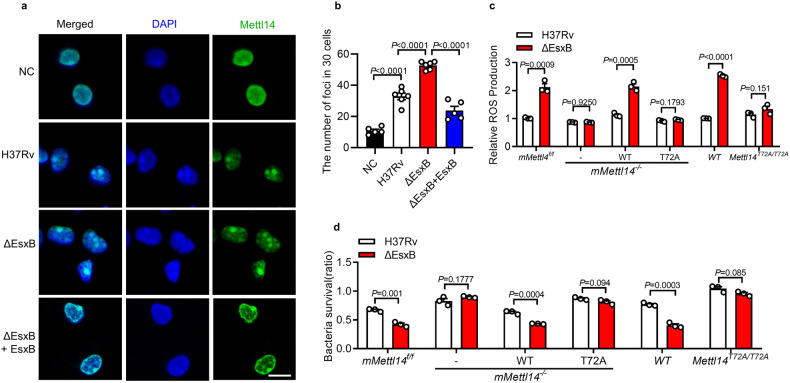
EsxB inhibits LLPS of METTL14. **a**, **b** Immunofluorescence staining for Mettl14 in mouse peritoneal macrophages infected with H37Rv, H37Rv(ΔEsxB) and H37Rv(ΔEsxB + EsxB) for 2 h (MOI = 5). Scale bar, 10 μm. Every point in **b** represents the number of Mettl14 foci in 30 cells. **c**, **d** The foci in the nucleus with a diameter over 200 nm were included. ROS assay (**c**) and bacterial survival ratio (CFU at 24 h/CFU at 2 h) (**d**) in *mMettl14f/f* or *mMettl14*^*−/−*^ macrophages transfected with vector (-) or plasmids encoding FLAG-tagged WT or T72A mutants of Mettl14, and WT or Mettl14^T72A^ macrophages infected with H37Rv or H37Rv(ΔEsxB) (MOI = 5) for 4 h in (**c**); 2 h and 24 h in (**d**). Results in (**a**) were repeated twice independently. Results in **c**, **d** reflect the mean ± SEM from at least three independent biological experiments. Two-tailed unpaired Student’s *t*-test were used.

Given that EsxB promotes the intracellular survival of *M. tuberculosis* via METTL14, we next investigated whether EsxB affects the LLPS of METTL14. We found that the deletion of EsxB markedly increased the formation of METTL14 condensates in *M. tuberculosis*-infected macrophages that reversed upon treatment with 1,6-HEX (Fig. 5a, b; Supplementary Fig. S5a–c). However, H37Rv and H37Rv△EsxB equally induced cytosolic foci of YTHDF2, another m^6^A binding protein that also undergoes LLPS in vitro and in vivo^64^(Supplementary Fig. S5d, e). These results suggest that EsxB inhibits LLPS specifically for METTL14. The complementation of *mMettl14*^*−/−*^ macrophages with the *Mettl14*^*T72A*^ mutant but not with *Mettl14* eliminated the inhibitory effect of EsxB on ROS production (Fig. 5c). EsxB did not enhance the intracellular survival of mycobacteria in *mMettl14*^*−/−*^ macrophages that were complemented with the *Mettl14*^*T72A*^ mutant that was seen in cells with *Mettl14* (Fig. 5d). Consistent with these findings, *Mettl14*^*T72A/T72A*^ macrophages infected with *M. tuberculosis* H37Rv:ΔEsxB did not exhibit the increase of ROS production (Fig. 5c) or decrease of bacteria survival (Fig. 5d) as in WT macrophages. Altogether, these results suggest that EsxB may inhibit the Thr72-dependent LLPS of METTL14, thereby suppressing ROS production to facilitate the intracellular survival of mycobacteria.

### EsxB inhibits p38-mediated phosphorylation of METTL14

**Fig. 6:**
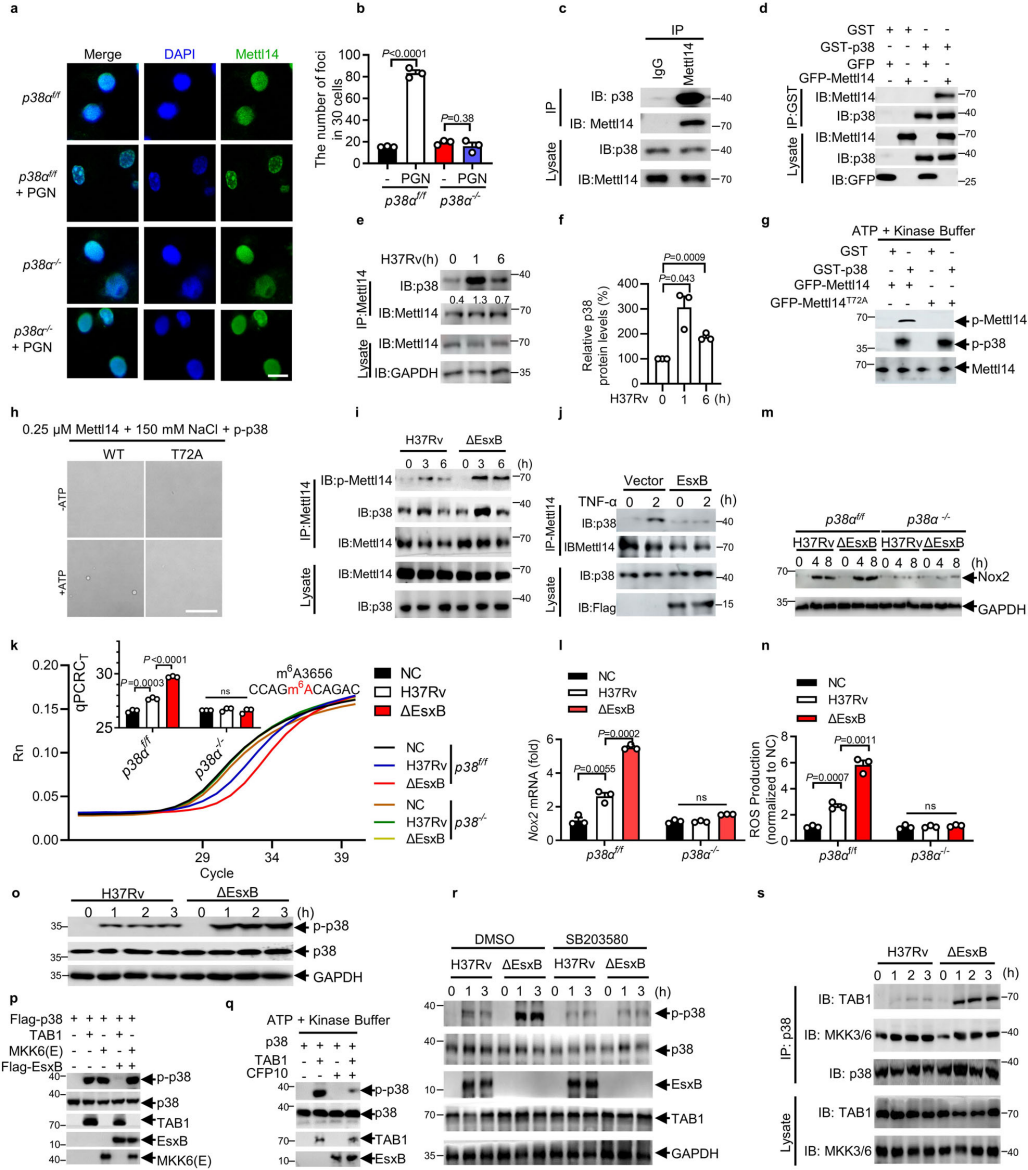
EsxB inhibits p38-mediated phosphorylation of METTL14. **a**, **b** Immunofluorescence staining for Mettl14 in peritoneal macrophages from *p38α*^floxp/floxp^ (*p38α*^*f/f*^) or *p38α*^floxp/floxp^; LysM-Cre (*p38α*^*−/−*^) mice stimulated with or without PGN for 2 h. Scale bar, 10 μm. Every point in **b** represents the number of Mettl14 foci in 30 cells. **c** Endogenous interaction of Mettl14 and p38. **d** Interaction of GST-p38 with GFP-METTL14 in vitro. **e**, **f** Endogenous interaction of Mettl14 and p38 in peritoneal macrophages infected with H37Rv for indicated times (MOI = 5). Relative gray intensity of p38 was shown in (**f**). **g** In vitro kinase assay of purified recombinant GST-p38 (active) with GFP-Mettl14-WT or GFP-Mettl14-T72A. **h** Mettl14-WT and Mettl14-T72A are subjected to in vitro kinase assay and then performed in vitro LLPS assay to test the ability of droplet formation. **i** IB and IP of mouse peritoneal macrophages infected with H37Rv or H37Rv(ΔEsxB) for indicated times (MOI = 5). **j** IB and IP of HEK293T cells transfected with plasmids encoding FLAG-EsxB for 24 h and stimulated with TNF-α for indicated times. **k**–**n** Real-time fluorescence amplification curves and bar plot of the threshold cycle (*C*_T_) of qPCR showing SELECT results for detecting m^6^A3656 in *Nox2* mRNA (**k**), qPCR analysis of *Nox2* mRNA (**l**), Nox2 protein levels (**m**) and changes in the levels of ROS (DCF staining; green) (**n**) in peritoneal macrophages from *p38α*^*f/f*^ or *p38α*^*−/−*^ mice infected with H37Rv or H37Rv(ΔEsxB) for 2 h in **k** or 4–8 h in (**l**–**n)** (MOI = 5). Rn is the raw fluorescence for the associated well normalized to the fluorescence of the passive reference dye (ROX). **o** IB analysis of protein levels from mouse macrophages infected with H37Rv or H37Rv(ΔEsxB) (MOI = 5) for indicated times. **p** IB analysis of iBMDM overexpression with Flag-p38 together with vector (-) or TAB1 or MKK6(E) or EsxB. **q** In vitro kinase assay of purified recombinant 6× His-p38 with TAB1 and EsxB. **r** IB analysis of mouse peritoneal macrophages treated with DMSO or SB203580 (10 μM) and infected with H37Rv or H37Rv(ΔEsxB) (MOI = 5) for indicated times. **s** IB and IP of mouse peritoneal macrophages infected with H37Rv or H37Rv(ΔEsxB) for indicated times (MOI = 5). All of the immunoblot data are representative images from one of three independent experiments. Results in **h** were repeated twice independently. Results in **b**, **f**, **k**, **l**, **n** reflect the mean ± SEM from three independent biological experiments. Two-tailed unpaired Student’s *t*-test were used.

We next investigated the possible upstream signaling that regulates the LLPS of METTL14. siRNA knockdown of p38α (p38) markedly reduced the peptidoglycan (PGN)-induced formation of METTL14 condensates (Supplementary Fig. S6a–c). Consistently, *p38*-deficient macrophages stimulated with PGN showed no increase in METTL14 LLPS, suggesting that *M. tuberculosis* infection may trigger the LLPS of METTL14 via *p38* (Fig. 6a, b; Supplementary Fig. S6c–e). Co-immunoprecipitation (Co-IP) analysis showed that endogenous p38 interacted with METTL14 (Fig. 6c). In an in vitro GST precipitation assay, purified recombinant GST-p38 was pulled down together with GFP-METTL14, indicating a direct interaction between p38 and METTL14 (Fig. 6d). Upon infection with *M. tuberculosis*, endogenous p38 was rapidly recruited to METTL14 in primary peritoneal macrophages (Fig. 6e, f), indicating a stimulus-dependent interaction between p38 and METTL14. Altogether, these results suggest that p38 may interact with METTL14 to promote the LLPS of METTL14.

Given that the LLPS of METTL14 depends on its Thr72 site and that p38 interacts with METTL14, we next investigated whether p38 phosphorylates METTL14 at Thr72. To further study the phosphorylation of METTL14 by p38, we generated a mouse monoclonal p-METTL14 antibody that was specific to the phosphorylation of the METTL14 Thr72 site (Supplementary Fig. S6f). In an in vitro kinase assay, the incubation of purified recombinant METTL14 with p38 led to the robust phosphorylation of METTL14 at Thr72, and the mutation of Thr72 markedly reduced the phosphorylation of METTL14 (Fig. 6g). Moreover, when incubated with p38 in vitro, we only observed phosphorylation and LLPS of WT METTL14 rather than T72A mutation (Fig. 6h; Supplementary Fig. S6g). These results suggest that p38 may mediate LLPS of METTL14 via phosphorylation of METTL14 at Thr72.

*M. tuberculosis* infection induced the phosphorylation of METTL14 at Thr72, indicated by the immunoblot analysis with the phosphorylation-specific antibody (Fig. 6i). Macrophages that were infected with the H37Rv:ΔEsxB strain exhibited much higher METTL14 phosphorylation compared with cells that were infected with WT H37Rv strains (Fig. 6i), suggesting that EsxB may inhibit the phosphorylation of METTL14. Given that p38 phosphorylates METTL14 at Thr72, we supposed that EsxB may target p38 or its upstream signal to inhibit the phosphorylation of METTL14. *M. tuberculosis* infection activates p38 via transforming growth factor-β (TGFβ)-activated kinase 1 (TAK1) in the Toll-like receptor (TLR) signaling pathway^65^. We found that EsxB interacted with p38, rather than TAK1 both in EsxB-expressing HEK293T cells or H37Rv-infected macrophages (Supplementary Fig. S6h, i). In HEK293T cells that overexpressed EsxB, p38 bound less with METTL14 (Fig. 6j). The deletion of EsxB from H37Rv enhanced the interaction between p38 and METTL14 in macrophages that were infected with H37Rv (Fig. 6i). In contrast, expression of EsxA in H37Rv:ΔEsxB-infected macrophages did not significantly change the inhibitory effect of EsxB on the interaction of p38 with METTL14 (Supplementary Fig. S6j). These data suggested that EsxB may interact with p38 and impede the interaction of p38 with METTL14, thus inhibiting the p38-mediated phosphorylation of METTL14.

It has been shown that p38 also directly interacts with and activates the AP-1 complex components ATF-2 and TCF (ELK4) to promote gene expression^66,67^. To further examine whether the inhibition of EsxB is specific for the action of p38 on METTL4, we examined the endogenous interaction between p38 with METTL14, ATF-2, and ELK4 in H37Rv- or H37Rv:ΔEsxB-infected peritoneal macrophages. The data showed that EsxB did not inhibit the interaction between p38 with ATF-2 and ELK4, suggesting that EsxB may specifically inhibit p38-mediated phosphorylation of METTL14 (Supplementary Fig. S6k). Moreover, the deletion of *p38* eliminated the inhibitory effect of EsxB on the m^6^A modification of *Nox2* mRNA in H37Rv-infected macrophages (Fig. 6k; Supplementary Fig. S6l). EsxB reduced *Nox2* mRNA levels, protein level, and ROS production in WT or control macrophages but not in p38 inhibitor-treated or *p38*^*−/−*^ macrophages (Fig. 6l–n; Supplementary Fig. S6m, n). These results suggest that EsxB may disrupt the interaction between METTL14 and p38, thereby inhibiting the p38-mediated phosphorylation of METTL14.

We found that EsxB markedly inhibited the phosphorylation of p38 in *M. tuberculosis* H37Rv-infected macrophages (Fig. 6o). Phosphorylation of p38 is regulated by its upstream Mitogen-activated protein kinase 3/6 (MKK3/6)-dependent phosphorylation of TGY residues on the active loop of p38 or TAB1-enhanced autophosphorylation of p38^66,68–71^. We found that EsxB inhibited the enhanced phosphorylation of p38 by TAB1 in HEK293T cells or in an in vitro kinase assay (Fig. 6p, q), but not by a dominant active MKK6 (MKK6(E)) mutant^69^ in HEK293T cells (Fig. 6p). Moreover, treatment with SB203580, a specific inhibitor of p38 autophosphorylation^69,72^, eliminated the inhibitory effect of EsxB on the p38 phosphorylation in *M. tuberculosis* H37Rv-infected macrophages (Fig. 6r). Finally, deletion of EsxB markedly increased the endogenous association of p38 with TAB1, but not with MKK3/6, in *M. tuberculosis* H37Rv-infected macrophages (Fig. 6s). Together, these results suggest that EsxB may disrupt the interaction of p38 with TAB1, thus inhibiting the TAB1-mediated autophosphorylation of p38.

### EsxB inhibits p38-mediated phosphorylation of METTL14 via Ser22

**Fig. 7:**
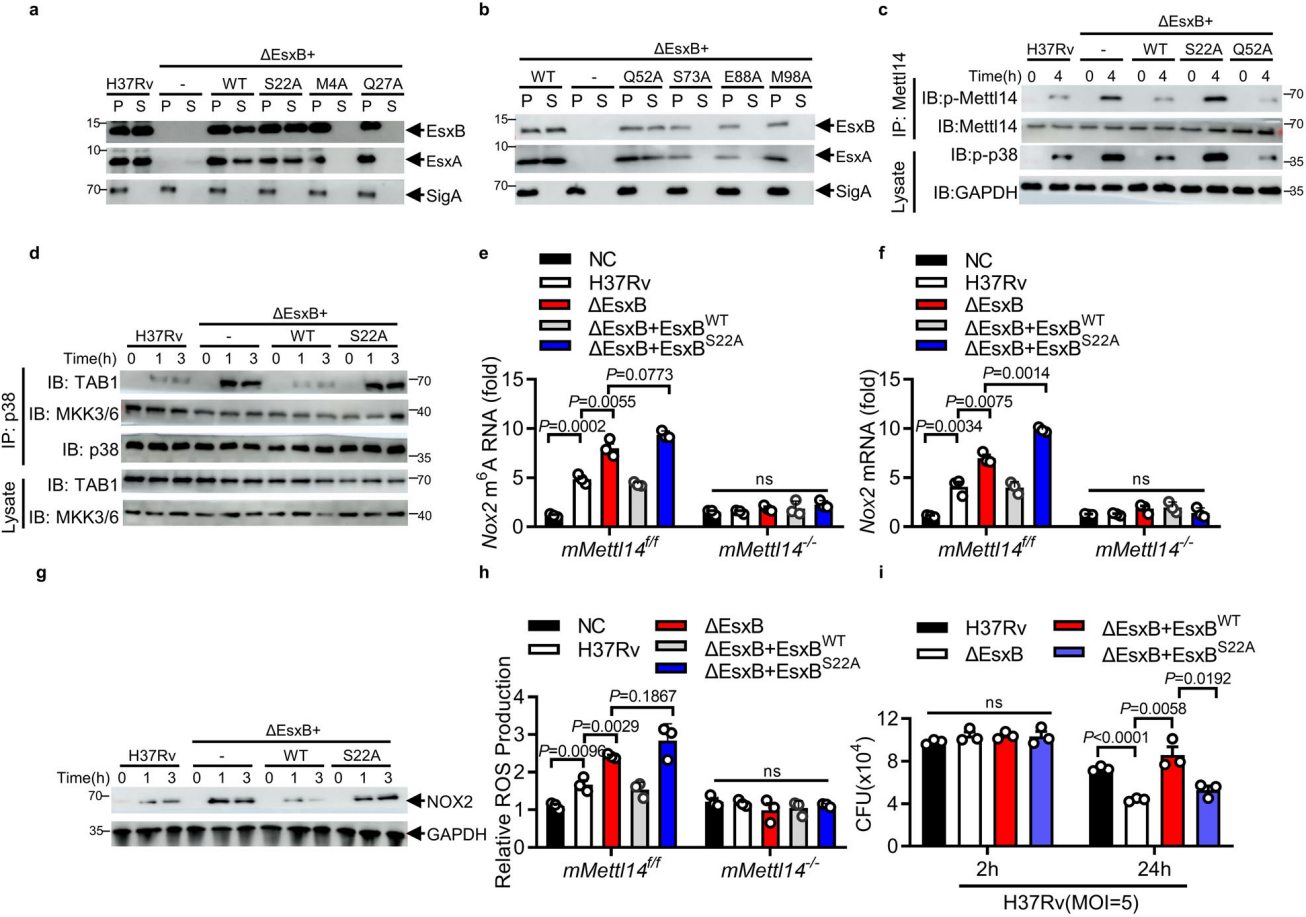
EsxB inhibits p38-mediated phosphorylation of METTL14 via Ser22. **a**, **b** IB of EsxA or EsxB in pellets and supernatant from H37Rv, H37RvΔEsxB, or H37Rv(ΔEsxB + EsxB^WT/mutants^) strains. **c** IB of mouse peritoneal macrophages infected with H37Rv, H37Rv(ΔEsxB) or H37Rv(ΔEsxB + EsxB^WT/S22A^) for indicated times (MOI = 5). **d** IB and IP of mouse peritoneal macrophages infected with H37Rv, H37Rv(ΔEsxB)or H37Rv(ΔEsxB + EsxB^WT/S22A^) for indicated times (MOI = 5). **e** MeRIP-qPCR analysis of relative m^6^A level of *Nox2* mRNA in peritoneal macrophages infected with H37Rv, H37Rv(ΔEsxB) or H37Rv (ΔEsxB + EsxB^WT/S22A^) for 2 h. **f** qPCR analysis of *Nox2* mRNA in mouse peritoneal macrophages following H37Rv, H37Rv(ΔEsxB) or H37Rv (ΔEsxB + EsxB^WT/S22A^) infection for 4 h (MOI = 5). **g** IB of mouse peritoneal macrophages infected with H37Rv, H37Rv(ΔEsxB) or H37Rv (ΔEsxB + EsxB^WT/S22A^) for indicated times (MOI = 5). **h** Changes in the levels of ROS (DCF staining; green) in mouse peritoneal macrophages following H37Rv, H37Rv(ΔEsxB) or H37Rv (ΔEsxB + EsxB^WT/S22A^) infection for 4 h (MOI = 5). **i** Peritoneal macrophages infected with H37Rv, H37Rv(ΔEsxB) or H37Rv (ΔEsxB + EsxB^WT/S22A^) for 2 h and 24 h, and then subjected to CFU assay (MOI = 5). Results in **a**–**d**, **g** are representative images from one of three independent experiments. All of the bar graphs in this figure reflect the mean ± SEM from three independent biological experiments. Two-tailed unpaired Student’s *t*-test were used.

It has been shown that the deletion of EsxB also abolishes EsxA secretion^46,47^ (Supplementary Fig. S2a). To further define the functional role of EsxB that is unrelated with its impact on EsxA, we attempted to identify the EsxB sites that do not affect EsxA secretion but are crucial for the inhibitory effect of EsxB on p38-mediated METTL14 phosphorylation. Based on the structure analysis of EsxB, we mutated seven EsxB sites that are predicted to be crucial for the function of EsxB^73^. Stable production of the EsxB (M4A), EsxB (S22A), EsxB (Q28A), EsxB (Q52A), EsxB (S73A), EsxB (E88A), and EsxB (M98A) mutant protein was detectable in H37Rv:ΔEsxB cell lysates, consistent with the dispensability of these residues for the production of EsxA (Fig. 7a, b). However, only EsxB (S22A) and EsxB (Q52A) mutants kept both EsxB and EsxA protein secretion (Fig. 7a, b). We further examined the function of EsxB (S22A) and EsxB (Q52A) mutants on the phosphorylation of the Mettl14 Thr72 site in *M. tuberculosis*-infected macrophages and found that H37Rv:ΔEsxB complemented with EsxB or EsxB (Q52A), but not with EsxB (S22A) restored the inhibition of Mettl14 Thr72 phosphorylation by *M. tuberculosis* infection (Fig. 7c). Moreover, Ser22 was found to be essential for EsxB to inhibit the interaction of p38 with TAB1, and subsequently TAB1-mediated autophosphorylation of p38 in *M. tuberculosis*-infected macrophages (Fig. 7c, d). Thus, our results suggest that EsxB may inhibit p38-mediated phosphorylation of METTL14 via Ser22.

Consistently, H37Rv:ΔEsxB or H37Rv:ΔEsxB+S22A mutant strains induced much higher m^6^A methylation (Fig. 7e), mRNA (Fig. 7f) and protein amount (Fig. 7g) of *Nox2* in *M. tuberculosis*-infected macrophages. H37Rv:ΔEsxB- or H37Rv:ΔEsxB + S22A mutant-infected macrophages exhibited higher ROS production (Fig. 7h) and lower bacteria survival (Fig. 7i). Thus, our data suggest that the inhibitory effect of ΔEsxB on the ROS production may require its interaction with p38 and TAB1, not through its interruption on the expression and secretion of ESAT-6.

### EsxB inhibits host anti-TB immunity via phosphorylation of METTL14

**Fig. 8:**
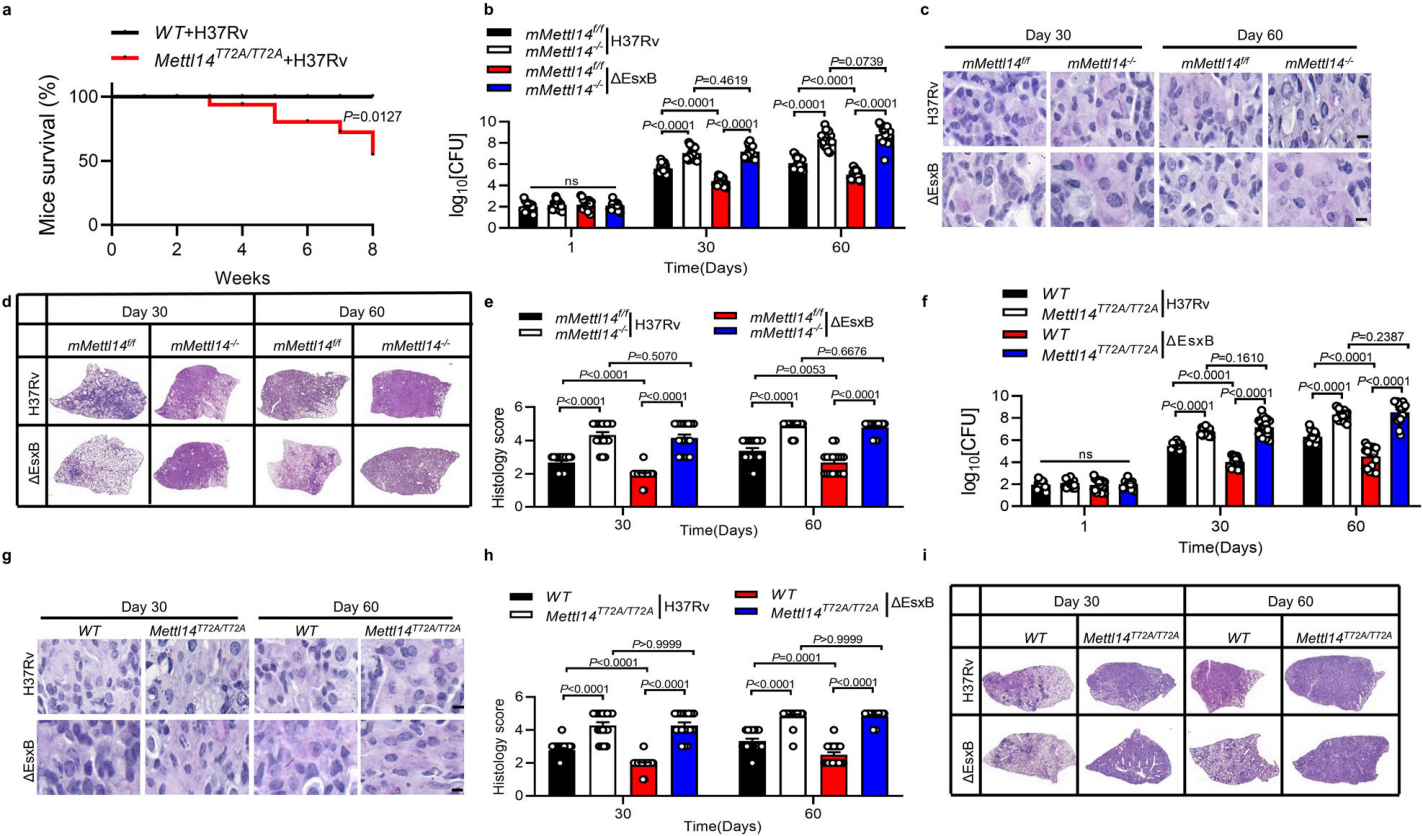
EsxB inhibits host anti-TB immunity via METTL14 Thr72. **a** Kaplan–Meier survival curves of mice infected with H37Rv for 8 weeks. Shown is the combined survival from two independent experiments (*n* = 18 mice per group). **b**–**e** 6–8-week-old female *mMettl14*^*f/f*^ or *mMettl14*^*−/−*^ mice were aerosol-infected with H37Rv (~ 200 CFUs per mouse). CFU of bacterial load at 1, 30 and 60 days post-infection (**b**); lung sections acid-fast staining (**c**), haematoxylin and eosin staining (**d**), and histology score (**e**). Scale bar, 1 mm. **f**–**i** 6–8-week-old female *WT* or *Mettl14*^*T72A/T72A*^ mice infected with H37Rv for 1, 30 and 60 days (~200 CFUs per mouse). We assayed: CFU of bacterial load at 1, 30 and 60 days post-infection (**f**); lung sections acid-fast staining (**g**), H&E staining (**h**), and histology score (**i**). Scale bar, 1 mm. Data in **b** and **f** show cumulative data from three independent experiments (mean ± SEM of *n* = 18) and a two-sided Mann–Whitney-*U*-test (**b**, **f**).

We next investigated the role of EsxB in Mettl14-dependent macrophage immunity during *M. tuberculosis* infection in *mMettl14*^*f/f*^ and *mMettl14*^*−/−*^ mice. Both male and female *mMettl14*^*−/−*^ mice exhibited normal growth rate and physiology relative to *mMettl14*^*f/f*^ counterparts, which is consistent with previous reports^74^. Notably, for *Mettl14*^*T72A/T72A*^ mice, at 8 weeks after infection, nearly 50% of mice died, but all the WT mice survived (Fig. 8a), suggesting that phosphorylation of METTL14 on T72 may promote the anti-TB immunity. Compared with *mMettl14*^*f/f*^ mice, *mMettl14*^*−/−*^ mice infected with *M. tuberculosis* exhibited a significant increase in bacterial burden (increased ~1.45 fold in log10 at day 30; increased ~2.23 fold in log10 at day 60) and histological damage in the lungs, supporting the essential role of METTL14 in host anti-TB immunity on controlling *M. tuberculosis* bacterial loads in vivo (Fig. 8b–e). In *mMettl14*^*f/f*^ mice, H37Rv:ΔEsxB markedly reduced *M. tuberculosis*-induced inflammatory infiltration and bacterial burden in the lungs. However, H37Rv and H37Rv:ΔEsxB caused comparable histopathological changes and bacterial burden in *mMettl14*^*−/−*^ mice (Fig. 8b–e). Our results suggest that EsxB is required by *M. tuberculosis* to evade host Mettl14-mediated immune responses.

To further examine the functional role of *Mettl14* Thr72 in the EsxB-mediated pathogenesis of *M. tuberculosis* infection in vivo, we challenged WT and *Mettl14*^*T72A/T72A*^ knock-in mice with H37Rv and H37Rv:ΔEsxB. As shown in Fig. 8f–i, *Mettl14*^*T72A/T72A*^ mice infected with *M. tuberculosis* exhibited a substantial increase in histological damage in the lungs than did their WT counterparts. Moreover, *Mettl14*^*T72A/T72A*^ mice displayed a significantly increased bacteria load in lung tissues from 30 and 60 days post-infection (increased ~1.3 fold in log10 at day 30; increased ~2.0 fold in log10 at day 60) (Fig. 8f–i). These results provided direct evidence that METTL14 Thr72 is crucial for host anti-TB immunity in vivo. Moreover, the mutation of Mettl14 on Thr72 in mice abolished the differences in histopathological changes and bacterial burden in the lungs of mice infected with H37Rv and H37Rv:ΔEsxB (Fig. 8f–i). Together, these data demonstrate that EsxB is required for the pathogenesis of *M. tuberculosis* via inhibiting phosphorylation of METTL14-mediated anti-TB immunity.

### METTL14 phosphorylation clinically associates with Nox2 mRNA level

Given that *M. tuberculosis*-secreted protein EsxB inhibits the p38 phosphorylation-dependent LLPS of METTL14, thereby reducing the m^6^A RNA methylation and mRNA stability of *Nox2*, which reduces ROS levels and increases intracellular survival of *M. tuberculosis* (Fig. 8a), we next investigated whether this regulatory mechanism or target is clinically associated with human tuberculosis caused by *M. tuberculosis*. We collected bronchoalveolar lavage fluid (BALF) cells from PTB (primary TB) or TB-negative patients and analyzed METTL14 T72 phosphorylation, *Nox2* mRNA, and ROS production. As shown in Supplementary Fig. S7a–c, BALF cells from PTB patients exhibited higher levels of METTL14 T72 phosphorylation, *Nox2* mRNA expression, and ROS production compared to TB-negative patients. These data suggest that METTL14 phosphorylation may be clinically associated with *Nox2* mRNA level and ROS production in TB patients.

## Discussion

The establishment of TB infection and disease development are largely determined by the interactions between host immune defense and immune evasion of *M. tuberculosis*^2–7^. m^6^A is the most abundant modification in mRNA and non-coding RNA in eukaryotic cells^16–19^. Our findings identify the mycobacterial secreted antigen EsxB as a previously unrecognized essential factor for the intracellular survival of *M. tuberculosis* through inhibiting METTL14-mediated m^6^A methylation of *Nox2* mRNA (Supplementary Fig. S7d). We found that *M. tuberculosis* infection leads to p38-mediated phosphorylation and subsequent LLPS of the m^6^A RNA “writer” METTL14, thereby enhancing the m^6^A methylation and stability of *Nox2* mRNA. However, the mycobacterial secreted protein EsxB inhibits TAB1-mediated autophosphorylation of p38 and downstream p38-mediated phosphorylation as well as LLPS of METTL14, thus reducing the m^6^A methylation *Nox2* mRNA and the production of ROS to promote the intracellular survival of *M. tuberculosis*. Thus, our findings not only indicate that m^6^A RNA modification plays a crucial role in the host defense against *M. tuberculosis* infection, but also reveal that EsxB appears to be a novel virulence factor that allows *M. tuberculosis* to employ an unexpected mechanism to evade host innate immunity. This mechanism depends on the reduced m^6^A mRNA modification of anti-mycobacterial gene transcripts by a mycobacterial secreted antigen.

Although m^6^A mRNA modification modulates epi-transcriptional regulation of gene expression, participating in multiple physiological and pathological processes^17,19,75–77^, whether m^6^A modifications regulate the immune responses against *M. tuberculosis* infection remains unknown. Our present study demonstrated that METTL14 is essential for limiting the intracellular growth of *M. tuberculosis* in macrophages. During *M. tuberculosis* infection, METTL14 promoted the m^6^A methylation of *Nox2* mRNA, which binds with IGF2BPs to increase the stability of *Nox2* mRNA in *M. tuberculosis*-infected primary macrophages. Increased production of NOX2 is responsible for the generation of ROS to limit the intracellular survival of mycobacteria^44^. Furthermore, the deletion of *Mettl14* dramatically impaired the anti-TB immunity as indicated by increased bacteria burden and pathological damage in the lung of *M. tuberculosis-*infected mice. Therefore, our results identified a novel role for m^6^A modification in innate immunity to *M. tuberculosis* infection, suggesting that m^6^A modification may be a potential target for the anti-TB drug or vaccine design.

Biomolecular condensates or droplets formed by LLPS are increasingly recognized as an important cellular phenomenon that is crucial for the regulation of human diseases^28–30^. It has been shown that the cytosolic m^6^A-binding proteins YTHDF1, YTHDF2, and YTHDF3 undergo LLPS, which can be further enhanced by multiple m^6^A modifications on RNA^64,78,79^. A more recent study indicated that the *S*-adenosylmethionine (SAM)-dependent LLPS of METTL3 dynamically regulates the assembly of the METTL3/METTL14/WTAP writer complex^80^. We found that *M. tuberculosis* infection induced the formation of METTL14 condensates through LLPS in macrophages. The formation of condensates through phase separation is often associated with low-complexity IDRs within RNA-binding proteins^81–83^. Moreover, phosphorylation within these disordered regions can control condensate assembly and disassembly^28,84,85^. Our present study demonstrated that LLPS of METTL14 depends on the phosphorylation of Thr72, a conserved site in the IDR of METTL14, by the p38 MAP kinase. A previous study identified S399 as one of the phosphorylation sites of METTL14, but no function of this site was specified^86,87^. Our findings demonstrate that phosphorylation of the Thr72 site by p38 is the first identified functional site required for METTL3–METTL14 complex formation and methylation activity, but further study is needed to clarify the functional consequences of p38-mediated phosphorylation of METTL14 on Thr72 by generating *Mettl14*^T72A^ knock-in mice. Future research will extend to the identification and characterization of the phosphorylation-dependent LLPS of m^6^A RNA modification regulators in other physiological and pathological processes.

*M. tuberculosis* is an extremely successful intracellular pathogen that can evade innate immune clearance to establish persistent infections inside macrophages^2–7^. It has been shown that p38 MAPK signaling pathways play critical roles in the *M. tuberculosis*-induced production of pro-inflammatory cytokines. Furthermore, p38 MAPK phosphorylation is related to inhibition of *M. tuberculosis* growth in macrophages. p38 MAPK is also associated with Th1 cell activation and differentiation, which is critical for protection against *M. tuberculosis*. Despite its crucial role in immune responses to *M. tuberculosis*, the mechanisms underlying the modulation of p38 MAP kinase remain unknown. p38 MAP kinase is activated by its upstream MKK3/6 or TAB1^66,68–71^. TAB1 interacts with p38 and promotes its autophosphorylation of p38^66,68–71^. Our data demonstrate that EsxB disrupts the interaction of p38 with TAB1, thus inhibiting the TAB1-mediated autophosphorylation of p38. Furthermore, our results showed that EsxB specifically inhibits the p38-mediated phosphorylation of METTL4 on T72A to impair ROS production and the clearance of *M. tuberculosis*. To the best of our knowledge, our study is the first to show the mechanism underlying the modulation of p38 signaling by *M. tuberculosis* to benefit its intracellular survival. However, given the numerous upstream regulators and downstream substrates of p38 MAPK relevant to the macrophage’s interaction with *M. tuberculosis*, more studies are needed to explore the regulation of p38 MAP kinase signaling during *M. tuberculosis* infection.

We found that deletion of EsxB from *M. tuberculosis* H37Rv enhanced the production of ROS in macrophages, which is consistent with previous reports that the addition of recombinant EsxB protein reduces the production of ROS and RNS in macrophages^14,15^. Although deletion of EsxB abolishes the secretion of EsxB and EsxA in *M. tuberculosis*, EsxA and EsxB have completely opposite functions in the regulation of ROS production that EsxA promotes^48–50^, while EsxB inhibits ROS production. In addition, expression of EsxA in iBMDM cells did not significantly change the enhanced effect of ΔEsxB on ROS production indicating that the observed effects of H37Rv:ΔEsxB on macrophages ROS production may primarily be mediated through EsxB deficiency but not EsxA deficiency. To test the specific role of EsxB-inhibited ROS production in H37Rv-infected macrophages, point mutations in EsxB allow substantial levels of secretion of EsxA, while abolished EsxB secretion is underway^88,89^. Mechanistically, our results showed that EsxB inhibits METTL14-mediated m^6^A methylation of *Nox2* mRNA to promote the intracellular survival of *M. tuberculosis*. To the best of our knowledge, our study is the first to show the immune evasion of *M. tuberculosis* at the m^6^A methylation level. However, we note that the inhibition by WT *M. tuberculosis* H37Rv on METTL14-mediated function is incomplete. Other METTL14-mediated functions may still contribute to the clearance of intracellular mycobacteria; thus, WT *M. tuberculosis* grows better when *Mettl14* is knocked out. Another report demonstrated that nuoG is an *M. tuberculosis* virulence factor that inhibits apoptosis via repressing ROS production in the phagosome, though the underlying mechanism remains to be determined^43^. We found that the intracellular survival of H37RvΔnuoG markedly decreased in *mMettl14*^*f/f*^ control macrophages, and the reduced survival of H37RvΔnuoG was not restored in *mMettl14*^*−/−*^ macrophages, suggesting that nuoG may repress ROS production to promote the intracellular survival of *M. tuberculosis* through other mechanisms, rather than METTL14-mediated *Nox2* m^6^A methylation. Whether *M. tuberculosis* modulates the m^6^A methylation of other anti-mycobacterial genes to trigger or inhibit the host immune responses will require further investigation.

It has been shown that NOX2 (also named CYBB) polymorphisms are significantly correlated with reduced risk of tuberculosis^90^ and CYBB missense mutations lead to recurrent BCG disease or recurrent tuberculosis^91–93^. By analyzing the blood gene expression profiles, Deng et al. found that CYBB mRNA is much higher in active pulmonary TB patients (PTB) compared to latent TB patients^94^. The SNPs of METTL14 rs62328061 GG genotype are significantly increased and the transcription levels of METTL14 were significantly decreased in PTB patients compared to normal controls^95^. In our study, we observed a clinical association of METTL14 phosphorylation with *Nox2* mRNA level and ROS production in PTB patients. Therefore, these findings that METTL14 and related gene expression levels are associated with tuberculosis infection and its severity in tuberculosis patients may provide more insights into the understanding of tuberculosis pathogenesis in humans.

Collectively, our findings provide evidence that m^6^A RNA modification plays a crucial role in the host immune defense against *M. tuberculosis* infection. Pathogenic mycobacteria evolved a secreted protein, EsxB, which is widely known as a specific antigen for the diagnosis of TB^96^, to suppress the m^6^A RNA modification and mRNA stability of anti-mycobacterial genes, thus highlighting the versatility of host–*M. tuberculosis* interactions (Supplementary Fig. S7). Our findings provide insights into the functional and regulatory mechanism of m^6^A RNA modification, raising the possibility of novel anti-tuberculosis treatments that target the EsxB–METTL4 interface.

## Materials and methods

### Bacterial strains and cells

Bacterial strains are described in Supplementary Table S1. Mycobacterial strains were grown in the following medium: Middlebrook 7H9(BD) broth supplemented with 0.05% Tween-80 (Sigma) and 10% oleic acid–albumin–dextrose–catalase (OADC) or Middlebrook 7H10 agar mixed with 10% OADC. For mycobacterial strain selection, the antibiotic hygromycin or kanamycin was mixed in a concentration of 100 μg/mL. Mycobacteria cultures were grown up to mid-log phase (an optical density at 600 nm of ~0.6). 7H10 solid medium preparation was mixed with 270 mL ddH_2_O, 5.7 g 7H10, and 1.5 mL glycerol, then, added penicillin (1:1000) and 30 mL OADC to culture tuberculosis from post-infected macrophages or mice.

HEK293T cells (ATCC CRL-3216) were resuspended in DMEM (HyClone) mixed with 10% (v/v) fetal bovine serum (FBS, Gibco) for experiments. Macrophages and THP1 cells (ATCC TIB-202) were cultured in RPMI-1640 medium supplemented with 10% (v/v) FBS. Peritoneal macrophages were obtained from mice (~6 weeks) three days after injection of thioglycollate (BD). All the cells were routinely tested for contamination by mycoplasma.

### Plasmids, reagents, and antibodies

Plasmids are described in Supplementary Table S1. The following antibodies and reagents were used for western blotting assay, immunofluorescence, or Co-IP: rabbit anti-GAPDH antibody (Catalog#/Clone: SAB2701826/polyclonal), rabbit anti-GFP antibody (Catalog#/Clone: AB10145/polyclonal), anti-METTL14 antibody (Catalog#/Clone: SAB5700855/ polyclonal), anti-METTL3 (Catalog#/Clone: AV34590) were purchased from Sigma-Aldrich; anti-EsxB antibody (Catalog#/Clone: ab45074) and Anti-ESAT6 antibody (Catalog#/Clone: ab45073) were from Abcam; anti-CYBB-NOX2 antibody (Catalog#/Clone: NBP2-38642/polyclonal) was from Novusbio; rabbit anti-phospho-p38 antibody (Catalog#/Clone: 9215/3D7), anti-p38 MAPK (Catalog#/Clone: 8690/D13E1), were from Cell Signaling Technology, Danvers, MA; anti-YTHDF2 antibody (Catalog#/Clone: 24744-1-AP/polyclonal) was purchased from Proteintech; mouse monoclonal antibody to Mettl14 phosphorylated at Thr72 (p-T72-METTL14) was generated by immunization of rabbits, in collaboration with AbClonal Biotech.

### Generation of complementary strain

H37Rv(ΔEsxB + GFP) and H37Rv(ΔEsxB + EsxB) strains were generated as a previous study^7^. Briefly, the shuttle vector pMV261 (provided by K. Mi, Institute of Microbiology, Beijing, China) was used to complement the strain H37RvΔEsxB with GFP or EsxB. Expression of EsxB in mycobacteria was examined by immunoblot analysis.

### Generation of mouse monoclonal p-METTL14 antibody

Mouse monoclonal antibody to Mettl14 phosphorylated at Thr72 (p-T72-METTL14) was generated by immunization of mice, in collaboration with AbClonal Biotech. Briefly, mice were immunized with a mixture of METTL14 Thr72 site by immunizing mice with the METTL14 peptide c(KLH)DEGE-pT-DEDK. c(KLH) is a keyhole limpet hemocyanin that fuses on the peptide through cysteine (“pT” indicates phosphorylated threonine) at a ratio of 1:1. Fusion screens: prepare homologous myeloma cells were mixed with mouse splenocytes in a certain proportion and the profusion agent polyethylene glycol was added. Under the action of polyethylene glycol, various lymphocytes can fuse with myeloma cells to form hybridoma cells. Fluent hybridoma cells were screened using HAT selective medium, laid on 10 plates (96-well plates), and ELISA assays. Subclonal screening: positive hybridoma cells obtained from the original well may be derived from two or more hybridoma cells. The clonal culture of hybridoma cells was carried out by the limited dilution method; each original well of fusion cells was laid on a plate, observed under an inverted microscope, marked wells with only a single clone growth, and the supernatant was taken for ELISA detection, and positive monoclonal cells were screened.

### Transfection and reverse transcription-PCR (RT-PCR) analysis

HEK293T cells were transiently transfected with Lipofectamine2000 (cn11668; Invitrogen). For siRNA experiments, the sequences of three siRNAs were designed to silence one specific target gene and peritoneal macrophages were transfected with a mixture of the three siRNAs using siRNA-Mate transfection reagent (G04003, from GenePharma), in accordance with the manufacturer’s instructions. A control siRNA (si-*Ctrl*: 5′-GGCUCUAGAAAAGCCUAUGCdTdT-3′) was used as a negative control. The three siRNA sequences are described in Supplementary Table S1. RNA preparation and quantitative PCR analysis were performed as previously described^97^.

### MeRIP-seq/qPCR

The RNA was extracted from infected cells, then fragmented to around 300–500 bp (covaris S220). The RNA was mixed with beads binding with NEB m^6^A antibody (E1610S) (A sample total RNA of 1 μg is taken as an example, which can be divided into two IP repeats). The mixture was divided into two parts at 4 °C for 4 h. The RNA binding on beads was washed with trizol and CHCl3 (1/5 volume), and added to the gel separation EP tube. The RNA was washed twice with 80% EtOH, 13,000 × *g*, 4 °C for 5 min, then dissolved in 10 μL of water, frozen at –80 °C. The RNA was used to qPCR or built into a library with SMARTer^®^ Stranded Total RNA-Seq Kit.

### SELECT detection

At least 3.0 × 10^7^ macrophages were collected after infection and the RNA was extracted from the collected cells. The SELECT detection was analyzed as previously described^63^. The RNA was mixed with 40 nM Up Primer, 40 nM Down Primer, and 5 μM dTTP (or dNTP) in 17 μL 1× CutSmart buffer (50 mM KAc, 20 mM Tris–HAc, 10 mM MgAc_2_, 100 μg/mL BSA, pH 7.9). The RNA and primers were annealed by incubating mixture at a temperature gradient: 90 °C for 1 min, 80 °C for 1 min, 70 °C for 1 min, 60 °C for 1 min, 50 °C for 1 min, and then 40 °C for 6 min. Subsequently, a 3 μL mixture containing 0.01 U Bst 2.0 DNA polymerase, 0.5 U SplintR ligase, and 10 nmol ATP was added in the former mixture to the final volume of 20 μL. The final reaction mixture was incubated at 40 °C for 20 min, denatured at 80 °C for 20 min, and kept at 4 °C. Afterwards, the qPCR reaction was performed in Applied Biosystems ViiATM7 Real-Time PCR System (Applied Biosystems, USA) or StepOnePlus™ Real-Time PCR System (Applied Biosystems). The 20 μL qPCR reaction was composed of 2× Hieff qPCR SYBR Green Master Mix (Yeasen) or PowerUp™ SYBR™ Green Master Mix (Applied Biosystems), 200 nM qPCRF primer, 200 nM qPCRR primer, 2 μL of the final reaction mixture and ddH2O. qPCR was run at the following condition: 95 °C, 5 min; (95 °C, 10 s; 60 °C, 35 s) × 40 cycles; 95 °C, 15 s; 60 °C, 1 min; 95 °C, 15 s (collect fluorescence at a ramping rate of 0.05 °C/s); 4 °C, hold. Data were analyzed by QuantStudioTM Real-Time PCR Software v1.3.

### RNA-seq and quantitative RT-PCR (RT-qPCR)

Total RNA was extracted using RNA-Quick Purification Kit (ES Science, Guangzhou, China) and cDNA was synthesized using the Prime-Script cDNA synthesis kits (Invitrogen, CA, USA) according to the manufacturer’s instructions. The reverse-transcribed cDNA products were used for qPCR analysis using the SYBR Green PCR kit (Invitrogen, California, USA).

### In vitro kinase assay

The recombinant human p38 alpha/MAPK14 protein (Active) (ab268832, abcam) was dissolved in kinase buffer (50 mM HEPES, pH 7.5, 10 mM MgCl_2_, 150 μM ATP, 50 mM NaCl, 0.02% BSA, and 1 mM DTT), then incubated with the recombinant protein METTL14-6 × His, METTL14^T72A^-6 × His, GFP-Mettl14 or GFP-METTL14^T72A^ (Sangon Biotech) were mixed with at 37 °C for 90 min. Then, the reaction mixture was subjected to LLPS assay or SDS-PAGE.

### Measurement of intracellular ROS levels

The intracellular ROS levels of macrophages were measured using a Reactive Oxygen Species Assay Kit (Beyotime Biotechnology, China); 2′,7′-dichlorofluorescein-diacetate (DCFH-DA), which is oxidized to fluorescent dichlorofluorescein (DCF) by intracellular ROS, is its principal component. The cells infected by different H37Rv strains were seeded in 96-well. Following the treatment, the cells were incubated with DCFH-DA for 20 min at 37 °C and then defined using fluorescence microscopy (Olympus) and measured at 488 nm excitation and 525 nm emission by a fluorescence spectrophotometer. Finally, the ROS levels could be quantified.

### Measurement of mito ROS

To detect mitochondrial superoxide mitoSOX (ThermoFitablesher) was used. Cells were stained for 10 min in 5 μM mitoSOX in 1× PBS 2% FBS at 37 °C. Image J software was used for image analysis. Mean fluorescence intensity was calculated from images by the Leica SP8 confocal microscopy system.

### RNA stability assay

For RNA stability assay, *Nox2* mRNA stability analysis in mouse peritoneal macrophages after 5 μg/mL actinomycin D (ActD) treatment. Peritoneal macrophages were transfected with siRNAs targeting *Mettl14* or *Igf2bp1* or a control sequence. 48 h later, a time course for RNA stability was started by infection, and then the transcription inhibitor (actinomycin D) was added. Cells were collected at the indicated timepoints. Expression levels were normalized to “0 h” and GAPDH was set as the reference gene.

### RNA half-life measurements

*Nox2* mRNA half-life was analyzed as previously described^98^. Mouse peritoneal macrophages were infected with different *M. tuberculosis* strains (MOI = 5) for 4 h and treated with actinomycin D (5 μg/mL). Cells were harvested at the indicated time points and analyzed via RT-PCR. *Nox2* mRNA levels were normalized to 0 h and *Gapdh* was used as a reference gene. *Nox2* mRNA half-life is calculated according to the following equation: ln(*C*_*i*_/*C*_0_) = −*kt*_*i*._ In this equation *k* is the degradation rate, *C*_*i*_ is the mRNA value at time *i*, and *t*_*i*_ is the time interval in hours. We first calculated the average degradation rate (*k*_a_) from each time point *k*_*i*_. The mRNA half-life *t*_1/2_ is ln(2)/*k*_a_.

### RIP-seq and RIP-qPCR

For RIP-seq, macrophages after infection were collected and then the pellet was resuspended in lysis buffer and rotated for 30 min at 4 °C. After cell lysis, the lysate was harvested by centrifugation at 12,000 × *g* for 10 min. The supernatant was transferred into a fresh 1.5 mL tube. Protease inhibitors and RNase inhibitors were added to the lysis buffer. About 10% volume of lysate was kept and the RNA was extracted as the input to detect the RNA integrity. The following RIP steps were performed by using an RNA-immunoprecipitation kit (EpibiotekTM, Cat#R1819). 40 μL of protein G beads were washed twice with IP buffer and added into the lysate together with the antibody, followed by incubation overnight at 4 °C. After incubation, the supernatant was transferred into a fresh 1.5 mL tube. The beads were recovered by magnet and resuspended within 1× wash buffer, rotated at 4 °C for 10 min. The supernatant was removed and the washing step was repeated three times. Co-precipitated RNA was extracted by TRIzol™ Reagent (Invitrogen™, Cat# 15596018) and Phenol-chloroform method. Co-precipitated RNA and input RNA were subjected to library construction by using EpiTM mini longRNA-seq kit (Epibiotek, Cat# E1802) according to the manufacturer’s protocols. Briefly, reverse transcription was performed using random primers, and the ribosome cDNA (cDNA fragments originating from rRNA molecules) was removed after cDNA synthesis using probes specific to mammalian rRNA. The directionality of the template-switching reaction not only preserves the 5’ end sequence information of RNA but also the strand orientation of the original RNA. Libraries for immunoprecipitated RNA were PCR amplified for 18 cycles. Library quality was determined using Qseq100 Bio-Fragment Analyzer (Bioptic Inc.). The strand-specific libraries were sequenced on the Illumina Novaseq 6000 system with paired-end 2 × 150 bp read length.

RIP-qPCR was analyzed as previously described^99^. At least 3.0 × 10^7^ macrophages were collected, was resuspended in 800 μL of lysis buffer (150 mM KCl, 20 mM Tris–HCl pH 7.5, 2 mM EDTA, 2 mM EDTA, 0.5% NP-40 0.5% Triton-100, 0.5 mM DTT, 1:100 protease inhibitor cocktail and 1:100 RNase inhibitor). Lysates were placed on ice for 15 min to remove cell debris. The lysates of 50 μL were kept for input. Remained lysates were mixed with anti-rabbit IgG magnetic beads (Thermo) and incubated with either 2 uL of anti-Mettl14 antibody or rabbit IgG overnight at 4 °C. Beads were washed with lysis buffer five times for 10 min. RNA was isolated from the beads by using trizol. RNAs were added with 1 μL glycogen and 1/10 volume, 3 M NaAc, and 1 volume isopropanol. The extracted RNAs were washed with 1 mL 75% ethanol once and added H_2_O to the pellet to elute the RNA, then reverse transcribed to cDNA for qPCR.

### In vitro LLPS assay

GFP-METTL14, GFP-METTL14^T72A^, METTL14-6 × His, METTL14^T72A^-6 × His and METTL14^T72D^-6 × His were purified from *E.coli* BL21. As for the LLPS of phosphorylated METTL14, METTL14-6 × His and METTL14^T72A^-6 × His were first phosphorylated via “in vitro kinase assay” as shown above. Then purified proteins or p-METTL14 were incubated in LLPS buffer (50 mM Tris, PH = 7.5, 10% (w/v) PEG 3550 (Sigma), 2 mM DTT and 0.1% BSA (w/v)) for 30 min at room temperature with or without 10% (w/v) 1,6-Hexanediol (Sigma). And then 5 μL of the sample was pipetted onto a coverslip and image using a Leica SP8 microscope with differential interference contrast (DIC).

### FRAP assay

The PFRP assay was performed using the FRAP module of the Leica SP8 confocal microscopy system. Briefly, GFP-METTL14 was bleached using a 488-nm laser beam. Bleaching was focused on a circular region of interest (ROI). After photobleaching, time-lapse images were captured. For each indicated time point (*t*), the fluorescence intensity within the bleached droplet was normalized to the fluorescence intensity of a nearby unbleached droplet. The normalized fluorescence intensity of pre-bleaching was set to 100%, and the normalized fluorescence intensity at each time point (It) was used to calculate the fluorescence recovery (FR) according to the following formula: FR(*t*) = It/*I* pre-bleaching. Image J was used for quantification and GraphPad Prism to plot and analyze the FRAP experiments.

### Flow cytometry

For analysis of macrophage markers of peritoneal macrophage from *mMettl14*^*f/f*^ and mMettl14^*−/−*^ mice, cells were blocked by anti-CD16/32 (Biolegend, S17011E) for 15 min at 4 °C firstly. Then anti-F4/80 (Biolegend, BM8), anti-CD86 (Biolegend, GL-1), and anti-MHC II (Invitrogen, M5/114.15.2) were added to the preincubated cell and incubated at 4 °C for 30 min. Next, all the cells were fixed and permeabilized by the BD Fixation/Permeabilization kit (BD, 554714) following the instructions. After incubation with anti-CD206 (Biolegend, C068C2), the peritoneal macrophages were washed 3 times with PBS and analyzed by CytoFLEX S (Beckman). The data were collected and analyzed by FlowJo™ (BD).

### In vitro infection of macrophages

For the in vitro infection of macrophages, non-adherent cells were removed by extensive washing with RPMI 1640. Bacteria were diluted and then incubated with cells for 2 h at 37 °C in 5% CO_2_. After washing twice, the infected cells were incubated in a fresh culture medium containing 50 μg/mL amikacin for 2 h. The infected cells were washed twice with PBS and lysed at 24 h with 0.1% (v/v) Triton X-100 in PBS. The number of viable intracellular bacteria (CFU) was determined by serial dilutions and plating out.

### Mice and infection

C57BL/6J*-Mettl14*^flox/flox^ mice were provided by Dr. Minghan Tong^100^ and were bred in SPF conditions at the Laboratory Animal Center of Tongji University. C57BL/6J-*p38α*^flox/flox^ mice provided by Dr. Jiahuai Han^101^. C57BL/6J-*Nox2*^*−/−*^ mice (Cat.# S-KO-01699) and C57BL/6J-*Lyz2*^*em1Cya*^ (*LysM*-Cre) (Cat.# S-KO-03032) mice were purchased from Cyagen. To obtain conditional Mettl14-KO mice in myeloid cells (for short as *mMettl14*^*−/−*^ in this study), 4-week-old *mMettl14*^*flox/flox*^ female mice were mated with male C57BL/6J-*Lyz2*^*em1Cy*a^ mice. Similarly, 4-week-old *p38α*^*flox/flox*^ female mice were mated with male C57BL/6J-*Lyz2*^*em1Cya*^ mice to obtain conditional P38-KO mice in myeloid cells. Moreover, all animal experiments were reviewed and approved by the Animal Experiment Administration Committee of Tongji University School of Medicine.

The procedure for the generation of Mettl14 T72A knockin mice is similar to previous report^102^. The T7 promoter sequence was fused with the Cas9 coding region which was cloned from pX260 plasmid (Addgene #42229). Similarly, the T7 promoter sequence and the targeting sequence of STING were fused to the guide RNA scaffold which was cloned from the pX330 plasmid (Addgene # 42230). In vitro transcription of Cas9 mRNA and sgRNAs targeting *Mettl14* was performed with mMESSAGE mMACHINE T7 ULTRA kit (ThermoFisher Scientific, AM1345, Waltham, MA) and MEGAshortscript T7 kit (ThermoFisher Scientific, AM1354, Waltham, MA) according to the manufacturer’s instructions, respectively. Both Cas9 mRNA and sgRNAs were then purified using MEGAclear Transcription Clean-Up Kit (Thermo Fisher Scientific, AM1908, Waltham, MA) and stored at −80 °C. One-cell embryos were collected from superovulated WT C57BL/6J female mice that had been mated with WT C57BL/6J male mice. Cas9 mRNA (50 ng/μL), sgRNA targeting *Mettl14* (50 ng/μL), and donor listed in Supplementary Table S1 (ssODN, 100 ng/μL) were mixed together and then injected into the one-cell embryos. The injected embryos were cultured in EmbryoMax KSOM Medium (Sigma-Aldrich, MR-106-D, USA) until the two-cell stage, followed by transferring into the oviducts of recipients at 0.5 dpc. Recipient female mice delivered pups at 19.5 dpc. The first generation of point-mutant mice was identified using the genotyping primers listed in Supplementary Table S1. The mice harboring the correct point mutation were crossed for the expansion of the mouse population.

Infection studies were carried out using a murine respiratory infection model. Six-week-old female mice were divided randomly into cages and infected with 100–200 CFU of different H37Rv strains using an aerosol method at the Biosafety Level-3 (BSL-3) Laboratory. At 28 days post-infection, the mice were killed, and bacterial counts in the lungs were determined by plating tenfold serial dilutions of each tissue homogenate on Middlebrook 7H10 agar plates. For histology, lung tissues from H37Rv-infected mice were fixed in 4% PFA and then stained with H&E or Ziehl–Neelsen stain (acid-fast). The stained slides were visualized by light microscopy.

### Histopathology analysis

An overall histology score was assigned to the lungs of mice based on the extent of granulomatous inflammation as follows: 0 = no lesion,1 = minimal lesion (1%–10% area of tissue in the section involved), 2 = mild lesion (11%–30% area involved); 3 = moderate lesion (30%–50% area involved); 4 = marked lesion (50%–80% area involved), and 5 = severe lesion (>80% area involved).

### Clinical samples, ethics approval, and consent to participate

All protocols were approved by the local ethics committee of Shanghai Pulmonary Hospital (permit number: K23-333Z), and signed informed consent was obtained from all subjects. All patients provided the written informed consent in accordance with the Declaration of Helsinki. Diagnosis of TB was based on clinical presentation and radiological findings (such as an X-ray or computed tomography (CT) scan) and was confirmed by a positive sputum culture. The test for the anti-human immunodeficiency virus (HIV) antibody was negative for all TB patients. The average age was 40 years, and 65% of the patients were male.

In TB-negative patients, the inclusion criteria were no history of previous TB or anti-mycobacterial treatments and no evidence of TB-related infiltration in chest X-rays. The average age was 35 years, and 60% of the controls were male.

### Quantification of Mettl14 foci

To quantify Mettl14 foci in the nucleus, the number of nuclear foci with a diameter over 200 nm was counted. The number of Mettl14 foci in 30 cells was shown as one point in every bar graph. About 100 cells were quantified in all of our studies.

### Statistical analysis

Statistical significance between groups was determined by two-tailed Student’s *t*-test, two-tailed analysis of variance followed by Bonferroni post hoc test or two-sided Mann–Whitney *U*-test. Differences were significant for *P* < 0.05. The experiments were not randomized, and the investigators were not blinded to allocation during experiments and outcome assessment.

### Supplementary information

Supplementary Information

## Data Availability

The main data supporting the findings of this study are available within the paper. Additional data are available from the corresponding authors upon reasonable request.
