# Supplementary material for: Mycobacterium tuberculosis inhibits METTL14-mediated m6A methylation of Nox2 mRNA and suppresses anti-TB immunity
Source: Cell Discov. 2024 Mar 29;10:36. doi: 10.1038/s41421-024-00653-4 (PMC10978938; doi:10.1038/s41421-024-00653-4)
Supplement: Supplementary file 1 — Supplementary Information [file 41421_2024_653_MOESM1_ESM.pdf]

Supplementary Figures

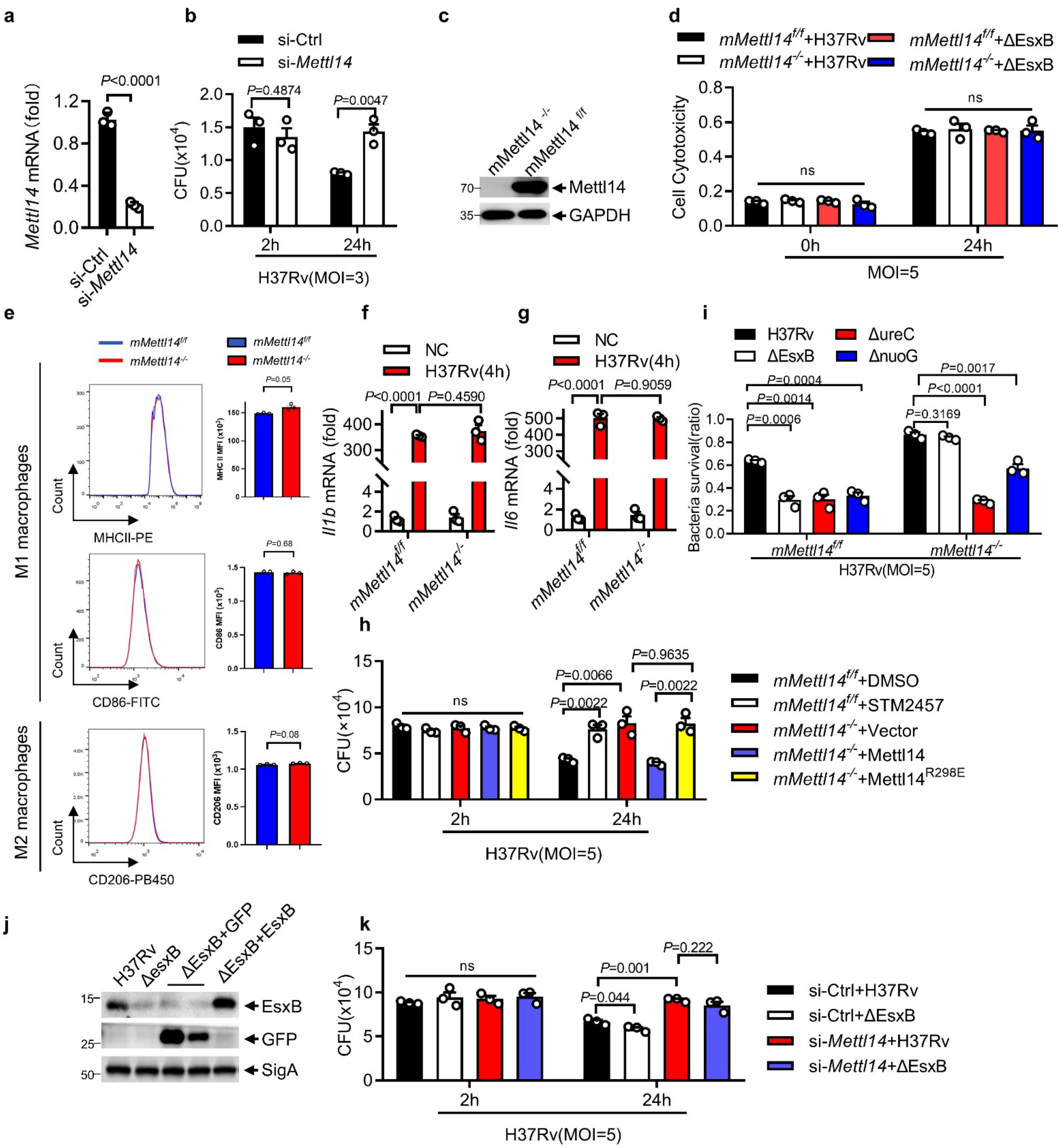

Supplementary Fig. S1 EsxB promotes survival of *M. tuberculosis* via METTL14.

(**a**) qPCR analysis of *Mettl14* mRNA level from control (si-*Ctrl*) and *Mettl14*-Konckdown (si-*Mettl14*) macrophages. **(b)** CFU assay in si-Ctrl or si-*Mettl14* mouse peritoneal macrophages infected with H37Rv for 2 h and 24 h, and then subjected to CFU assay (MOI = 5). (**c**) Immunoblot (IB) of Mettl14 in peritoneal macrophages isolated form Mettl14$^{f/f}$ or mMettl14$^{-/-}$ mice. (**d**) *mMettl14*$^{f/f}$ or *mMettl14*$^{-/-}$ mouse peritoneal macrophages infected with H37RvΔEsxB for indicated times (MOI = 5), and then test the cell viability via

LDH assay. (**e**) *mMettl14*$^{f/f}$ or *mMettl14*$^{-/-}$ mouse peritoneal macrophages were isolated and detected the MHC-II and CD86 (M1 macrophages marker) and CD206 (M2 macrophages marker) by FACS. (**f, g**) qPCR analysis the *Il1b* and *Il6* mRNA levels in *mMettl14*$^{f/f}$ or *mMettl14*$^{-/-}$ mouse peritoneal macrophages infected with H37Rv and H37RvΔEsxB for 4 h (MOI = 5). (**h**) CFU assay and bacteria survival of *mMettl14*$^{f/f}$ macrophages treated with or without STM2457 (2 μM); or *mMettl14*$^{-/-}$ peritoneal macrophages transfected with vector, HA-Mettl14 and HA-Mettl14-R298E and infected with H37Rv (MOI = 5) for 2 h and 24 h. (**i**) Bacteria survival analysis of *mMettl14*$^{f/f}$ or *mMettl14*$^{-/-}$ peritoneal macrophages infected with H37Rv, H37Rv(ΔEsxB), H37Rv(ΔnuoG) or H37Rv(ΔureC) (MOI = 5) for 2 h and 24 h. The bacterial survival ratio is CFU at 24 h/ CFU at 2 h. (**j**) IB of EsxB in H37Rv, H37Rv(ΔEsxB), H37Rv(ΔEsxB+GFP) and H37Rv(ΔEsxB+EsxB) strains. (**k**) Si-Ctrl and si-*Mettl14* mouse peritoneal macrophages infected with H37Rv or H37Rv(ΔEsxB) for 2 h and 24 h, and then subjected to CFU assay (MOI = 5). All of the immunoblot data are representative images from one of three independent experiments. All of bar graphs of this figure reflect the mean ± s.e.m from three independent biological experiments. Two-tailed unpaired Student's t-test were used.

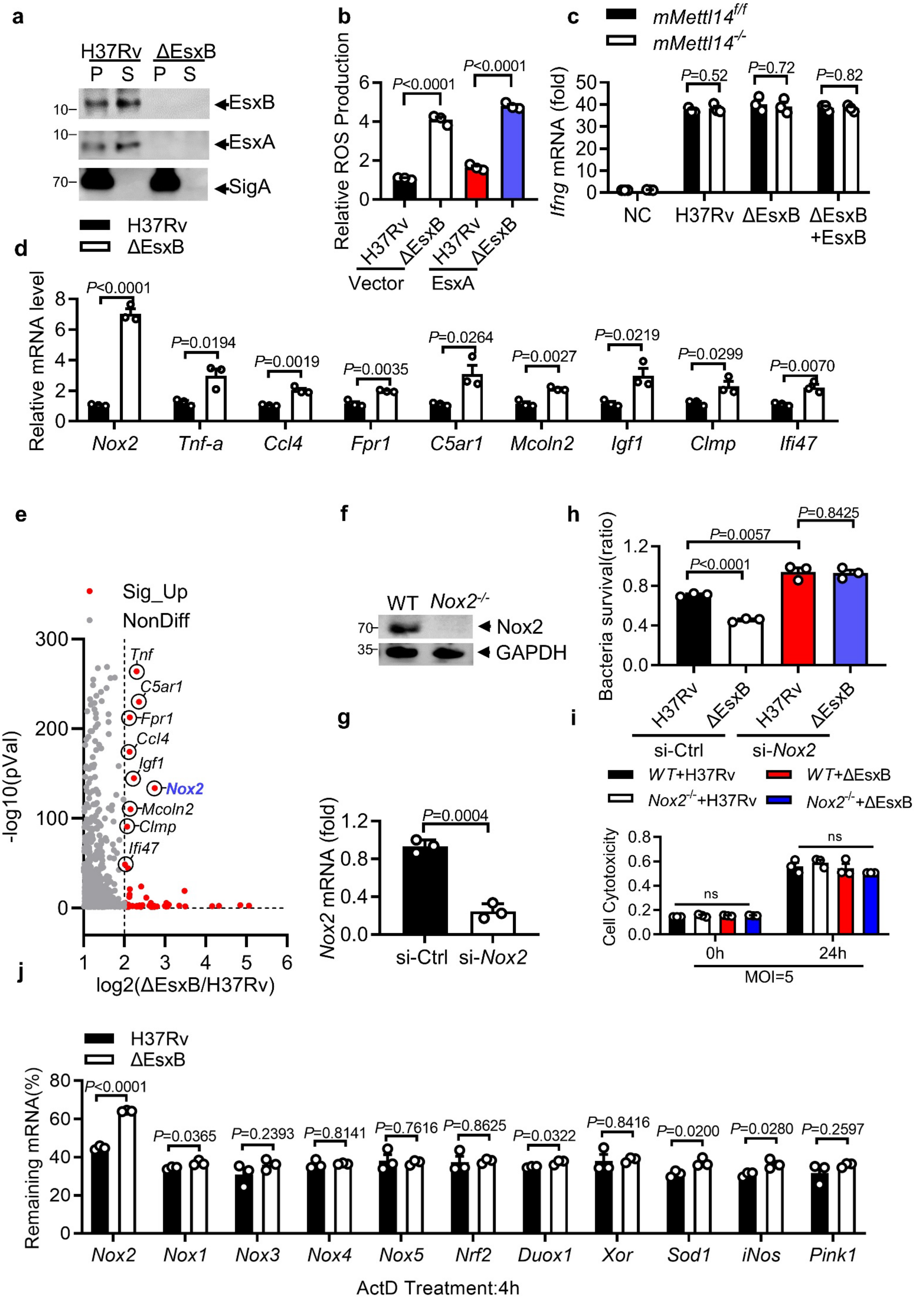

Supplementary Fig. S2 EsxB inhibits the ROS via disrupting Nox2 mRNA stability

(**a**) IB of EsxA and EsxB in pellets and supernatant from H37Rv and H37RvΔEsxB strain. (**b**) iBMDM cell were transfected with vector or Flag-EsxA , then infected with H37Rv or H37Rv(ΔEsxB) for 4 h and test ROS level. (**c**) *Ifng* relative mRNA levels were detected in *mMettl14*$^{f/f}$ or *mMettl14*$^{-/-}$ mouse peritoneal macrophages which were infected with H37Rv, H37Rv(ΔEsxB), and H37Rv(ΔEsxB+EsxB) for 4 h. (**d**) qPCR analysis of mRNA levels from mouse peritoneal macrophages infected with H37Rv or H37Rv(ΔEsxB) for 4 h (**e**) Volcano plot shows the differentially expressed genes identified from RNA-seq analysis of mouse peritoneal macrophages infected with H37Rv or H37Rv(ΔEsxB). (**f**) IB of Nox2 in peritoneal macrophages from WT or *Nox2*$^{-/-}$ mice. (**g**) qPCR analysis of *Nox2* mRNA level from si-Ctrl and si-*Nox2* mouse peritoneal macrophages. (**h**) Bacteria survival ratio (24 h CFU/ 2 h CFU) is shown in si-Ctrl and si-*Nox2* mouse peritoneal macrophages infected with H37Rv and H37RvΔEsxB (MOI = 5). (**i**) WT or *Nox2*$^{-/-}$ mouse peritoneal macrophages infected with H37Rv or H37RvΔEsxB for indicated times (MOI = 5), then test the cell viability via LDH assay. (**j**) mRNA of ROS or RNS related genes analysis in macrophages after actinomycin D treatment. Mouse peritoneal macrophages were infected with H37Rv, H37RvΔEsxB (MOI = 5) for 4 h and treated with actinomycin D (5 μg/mL). All of the immunoblot data are representative images from one of three independent experiments. All of bar graphs of this figure reflect the mean ± s.e.m from three independent biological experiments. Two-tailed unpaired Student's t-test were used.

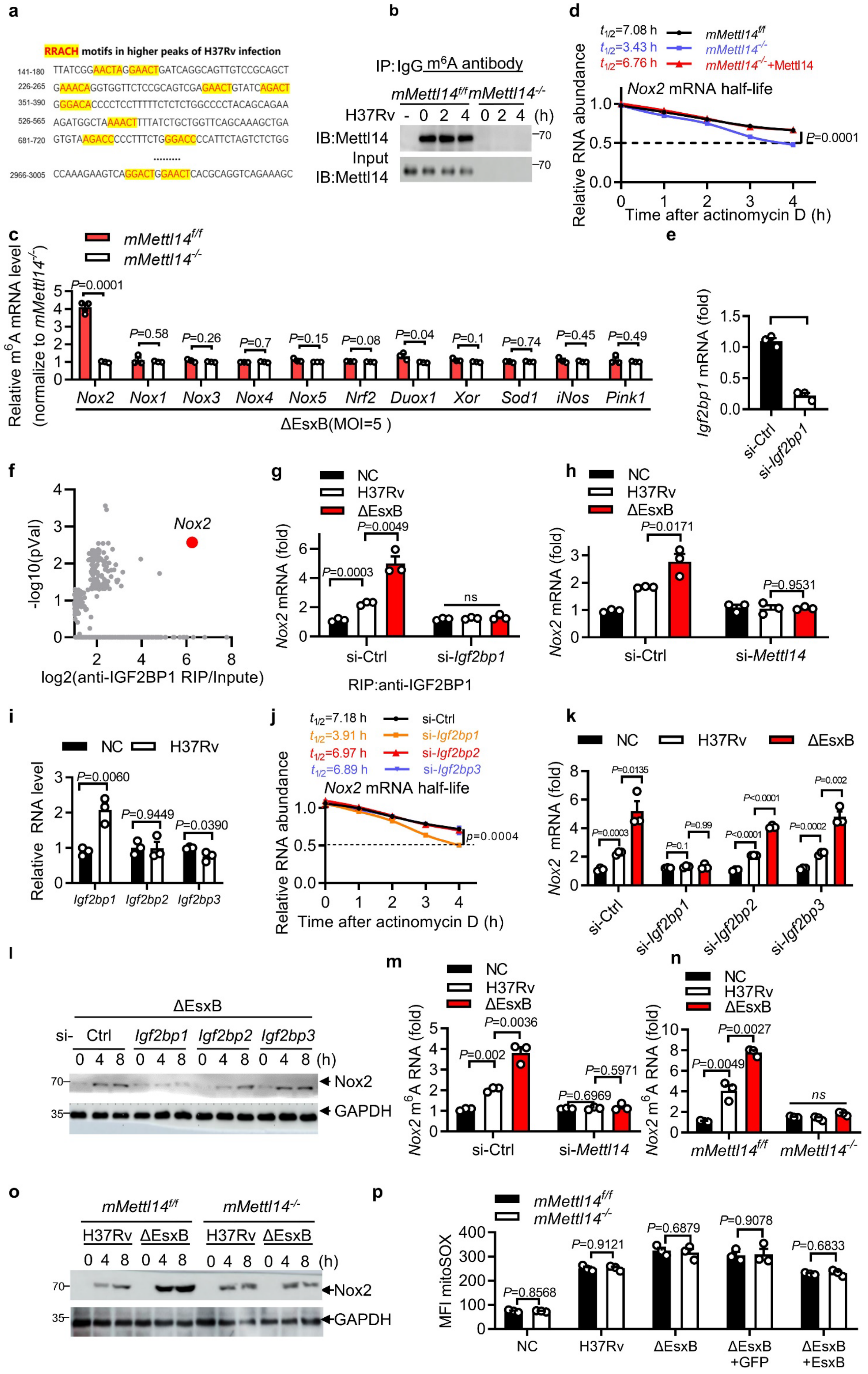
a
RRACH motifs in higher peaks of H37Rv infection
141-180 TTATCGGAACTAGGAACTGATCAGGCAGTTGTCCGCAGCT
226-265 GAAACAGGTGGTTCTCCGCAGTCGAGAACTGTATCAGACT
351-390 GGGACACCCCTCCTTTTTCTCTCTGGCCCCTACAGCAGAA
526-565 AGATGGCTAAAACTTTTATCTGCTGGTTCAGCAAAGCTGA
681-720 GTGTAAGACCCCCTTTCTGGGACCCCATTCAGTCTCTGG
....
2966-3005 CCAAAGAAGTCAGGACTGGAACTCACGCAGGTCAGAAAGC
b
IP: IgG m6A antibody
mMettl14f/f mMettl14-/-
H37Rv - 0 2 4 0 2 4 (h)
IB:Mettl14
Input
IB:Mettl14
–70
–70
d
t1/2=7.08 h mMettl14f/f
t1/2=3.43 h mMettl14-/-
t1/2=6.76 h mMettl14-/-+Mettl14
Nox2 mRNA half-life
Relative RNA abundance
P=0.0001
Time after actinomycin D (h)
c
mMettl14f/f
mMettl14-/-
Relative m6A mRNA level (normalize to mMettl14-/-)
P=0.0001 P=0.58 P=0.26 P=0.7 P=0.15 P=0.08 P=0.04 P=0.1 P=0.74 P=0.45 P=0.49
Nox2 Nox1 Nox3 Nox4 Nox5 Nrf2 Duox1 Xor Sod1 iNos Pink1
ΔEsxB(MOI=5)
e
Igf2bp1 mRNA (fold)
si-Ctrl si-Igf2bp1
f
Nox2
-log10(pVal)
log2(anti-IGF2BP1 RIP/Inpute)
g
NC
H37Rv
ΔEsxB
P=0.0049
P=0.0003
ns
Nox2 mRNA (fold)
si-Ctrl si-Igf2bp1
RIP:anti-IGF2BP1
h
NC
H37Rv
ΔEsxB
P=0.0171
P=0.9531
Nox2 mRNA (fold)
si-Ctrl si-Mettl14
i
NC H37Rv
Relative RNA level
P=0.0060 P=0.9449 P=0.0390
Igf2bp1 Igf2bp2 Igf2bp3
j
t1/2=7.18 h si-Ctrl
t1/2=3.91 h si-Igf2bp1
t1/2=6.97 h si-Igf2bp2
t1/2=6.89 h si-Igf2bp3
Nox2 mRNA half-life
Relative RNA abundance
p=0.0004
Time after actinomycin D (h)
k
NC H37Rv ΔEsxB
Nox2 mRNA (fold)
P=0.0135 P=0.0003 P=0.99 P=0.1 P<0.0001 P<0.0001 P=0.002 P=0.0002
si-Ctrl si-Igf2bp1 si-Igf2bp2 si-Igf2bp3
l
ΔEsxB
si- Ctrl Igf2bp1 Igf2bp2 Igf2bp3
0 4 8 0 4 8 0 4 8 0 4 8 (h)
70– Nox2
35– GAPDH
m
NC
H37Rv
ΔEsxB
P=0.0036 P=0.002 P=0.5971 P=0.6969
Nox2 m6A RNA (fold)
si-Ctrl si-Mettl14
n
NC
H37Rv
ΔEsxB
P=0.0027 P=0.0049 ns
Nox2 m6A RNA (fold)
mMettl14f/f mMettl14-/-
o
mMettl14f/f mMettl14-/-
H37Rv ΔEsxB H37Rv ΔEsxB
0 4 8 0 4 8 0 4 8 0 4 8 (h)
70– Nox2
35– GAPDH
p
mMettl14f/f
mMettl14-/-
MFI mitoSOX
P=0.8568 P=0.9121 P=0.6879 P=0.9078 P=0.6833
NC H37Rv ΔEsxB ΔEsxB +GFP ΔEsxB +EsxB

Supplementary Fig. S3 EsxB inhibits m^{6}A methylation of *Nox2* mRNA via METTL14.

(**a**) Alignment between 3′ UTRs exon of higher peaks in H37Rv infected sequences. Specific RRACH motifs are indicated in red, respectively. (**b**) IP-m^{6}A and IB-Mettl14 in *mMettl14*$^{f/f}$ and *mMettl14*$^{-/-}$ macrophages infected with H37Rv for 0, 2, 4 h (MOI = 5). (**c**) m6A mRNA levels of ROS related genes were analyzed in macrophages. *mMettl14*$^{f/f}$ and *mMettl14*$^{-/-}$ mouse peritoneal macrophages were infected with H37RvΔEsxB (MOI = 5) for 2 h. m^{6}A mRNA levels of each gene in *mMettl14*$^{f/f}$ macrophages were normalized to the results in *mMettl14*$^{-/-}$ macrophages. (**d**) *Nox2* mRNA stability analysis in *mMettl14*$^{f/f}$ or *mMettl14*$^{-/-}$ macrophages transfected with vector or plasmids encoding FLAG-tagged WT Mettl14 and infected with H37Rv(ΔEsxB) for 4 h following actinomycin D treatment for indicated times. (**e**) qPCR analysis of *Igf2bp1* mRNA level from si-Ctrl and si-*Igf2bp1* mouse peritoneal macrophages. (**f**) Volcano plot shows IGF2BP1-bound mRNAs infected with H37Rv(ΔEsxB) for 4 h. x axis shows log-fold change and y axis shows p value for corresponding binding-mRNA. Representative enriched mRNA (NOX2) is highlighted in red. (**g**) MeRIP-qPCR analysis of relative m6A Nox2 mRNA in si-Ctrl or si- *Igf2bp1* macrophages infected with H37Rv for 4 h. (**h**) *Nox2* mRNA levels in si-Ctrl and si-*Mettl14* macrophages infected with H37Rv or H37Rv(ΔEsxB) for 4 h. (**i**)qPCR analysis of Igf2bp1,2 and 3 mRNA levels from mouse peritoneal macrophages. (**j**) Nox2 mRNA stability analysis in si-Ctrl or si- *Igf2bp1,2* or *3* macrophages infected with H37Rv(ΔEsxB) for 4 h (MOI = 5) before treated with actinomycin D (5 µg/mL). Expression levels were normalized to 0h and *Gapdh* was used as reference gene. (**k**) *Nox2* mRNA levels in si-Ctrl or si- *Igf2bp1,2* or *3* macrophages infected with H37Rv or H37Rv(ΔEsxB) for 4 h (MOI = 5). (**l**) IB analysis of Nox2 protein levels from si-Ctrl or si- *Igf2bp1,2* or *3*mice macrophages infected with H37Rv(ΔEsxB) (MOI = 5) for 0, 4, 8 h. (**m**) *Nox2* m^{6}A mRNA levels in si-Ctrl and si-*Mettl14* macrophages infected with H37Rv or H37Rv(ΔEsxB) for 4 h. (**n**) MeRIP-qPCR analysis of relative m^{6}A *Nox2* mRNA in *mMettl14*$^{f/f}$ and *mMettl14*$^{-/-}$ mouse peritoneal macrophages infected with H37Rv and H37RvΔEsxB for 2 h (MOI = 5). (**o**) IB analysis of Nox2 protein levels from *mMettl14*$^{f/f}$ and *mMettl14*$^{-/-}$ mouse macrophages infected with H37Rv or H37Rv(ΔEsxB) (MOI = 5) for 0, 4, 8 h. (**p**) Mitochondrial ROS levels were detected in *mMettl14*$^{f/f}$ or *mMettl14*$^{-/-}$ mouse peritoneal macrophages infected with H37Rv, H37Rv(ΔEsxB), and H37Rv(ΔEsxB+GFP) and H37Rv(ΔEsxB+EsxB) for 4 h (MOI = 5). Results in **b, l** and **o** are representative images from one of three independent experiments. All of the data (except **a, b, f, l** and **o**) in this figure reflect the mean ± s.e.m from three independent biological experiments. Two-tailed unpaired Student's t-test were used.

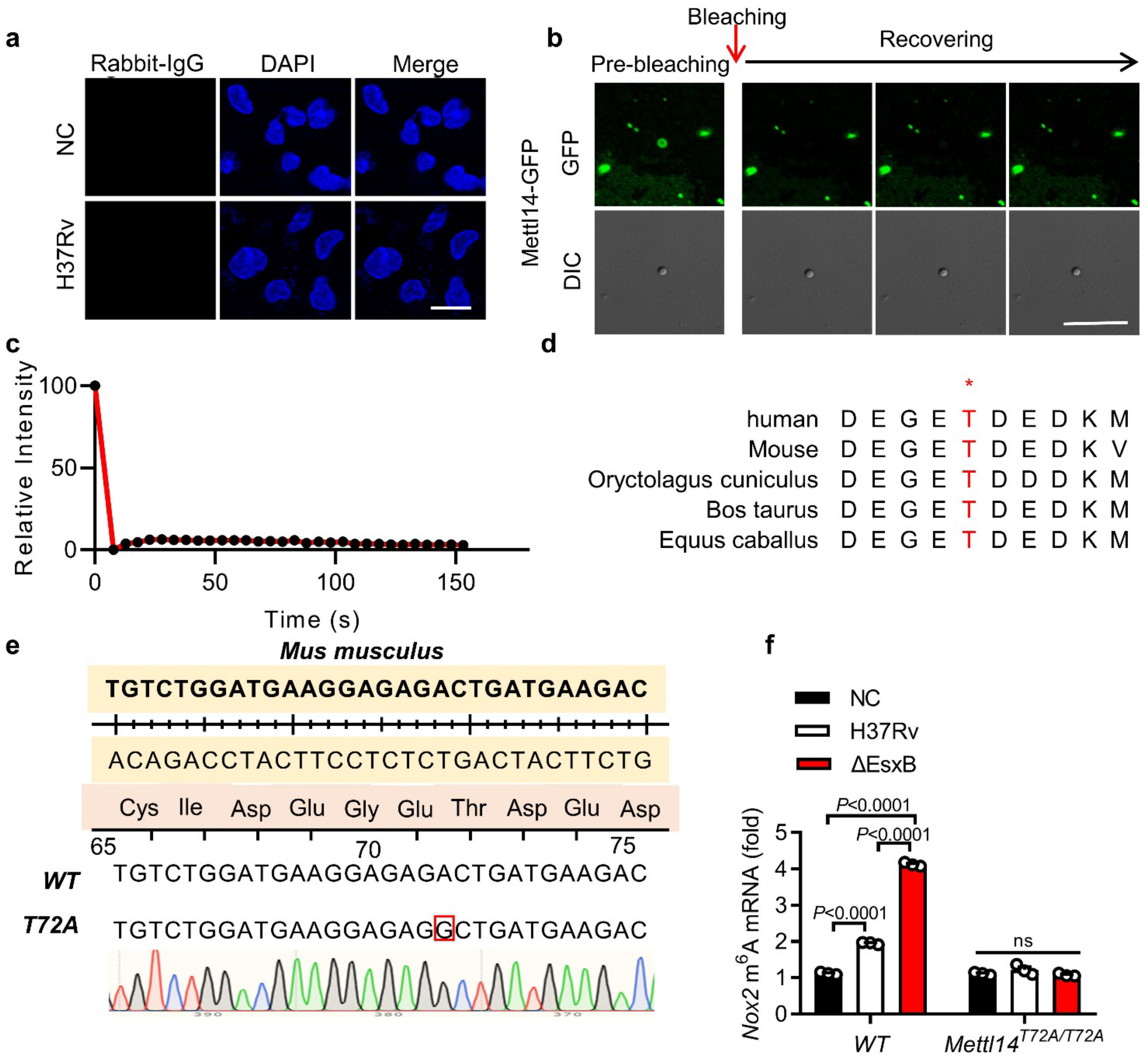

Supplementary Fig. S4 LLPS of METTL14 via Thr72.

(**a**) Immunofluorescence staining for IgG in mouse peritoneal macrophages infected with H37Rv for 2 h (MOI = 5), Scale bar, 10 μm. (**b**) Representative images of the FRAP analysis of METTL14-GFP in vitro. Scale bar, 5 μm. (**c**) The FRAP recovery curve by averaging the signals 8 droplets with similar sizes after photobleaching. The relative fluorescence intensity of each droplet prior to photobleaching was set to 100%. (**d**) Alignment of Mettl14 in different kinds of mammals. The site of T72 was indicated in red. **(e)** The genotyping results show the obtainment of *Mettl14*$^{T72A/T72A}$ knock-in mice. (**f**) MeRIP-qPCR analysis of relative m^{6}A *Nox2* mRNA from *WT* or *Mettl14*$^{T72A/T72A}$ mice infected with H37Rv(ΔEsxB) for 2 h (MOI = 5), mean ± s.e.m from three independent biological experiments, Two-tailed unpaired Student's t-test were used.

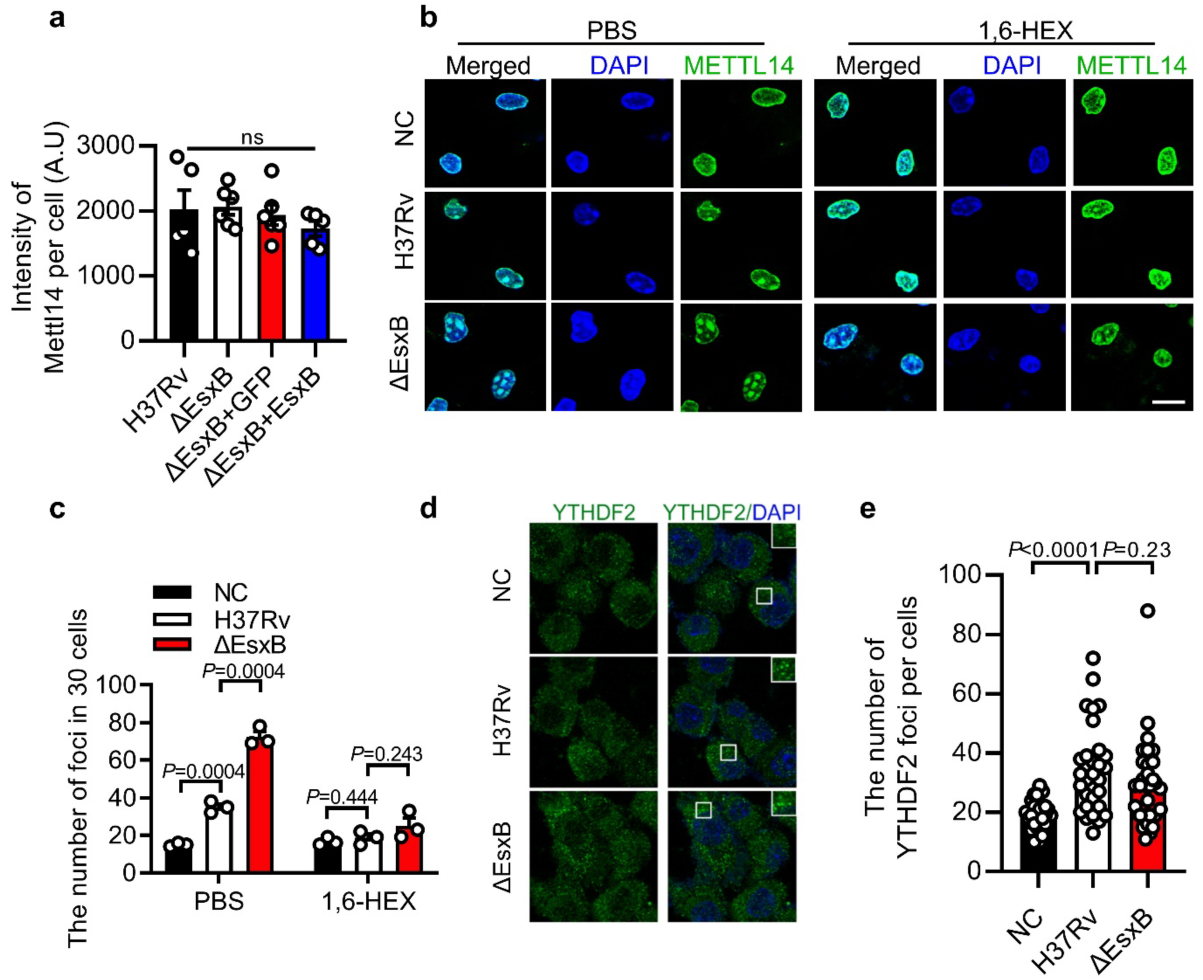

Supplementary Fig. S5 EsxB inhibit LLPS of METTL14 specifically.

(a) Analysis of intensity of Mettl14 foci in mouse peritoneal macrophages infected with H37Rv, H37Rv(ΔEsxB) and H37Rv(ΔEsxB+EsxB) for 2 h by Image J. Related to Fig. 5a. **(b, c)** Immunofluorescence staining for Mettl14 in mouse peritoneal macrophages infected with H37Rv, H37Rv(ΔEsxB) for 2 h (MOI = 5), the cell was whether treated with PBS or 10% 1,6- hexanediol (1,6-HEX) for 10 min. Every point in (**c**) represents the number of Mettl14 foci in 30 cells. Scale bar, 10 μm. The foci in the nucleus with a diameter over 200 nm were included. (**d, e**) Immunofluorescence staining for YTDHF2 in mouse peritoneal macrophages infected with H37Rv, H37Rv(ΔEsxB) for 2 h (MOI = 5). Every point in (**e**) represent the number of YTHDF2 foci per cells. Scale bar, 10 μm. Two-tailed unpaired Student's t-test were used.

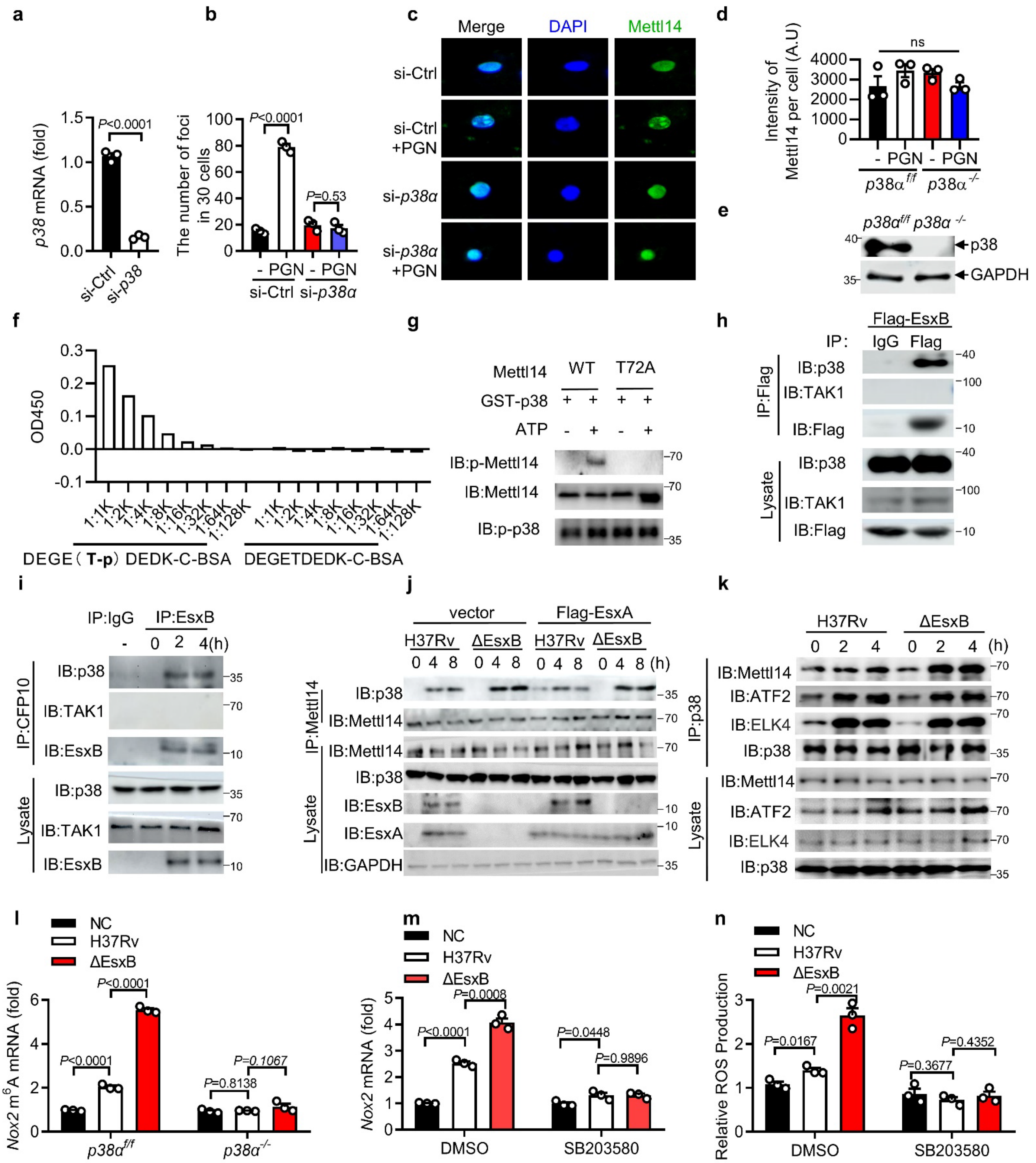

Supplementary Fig. S6 EsxB disrupts the interaction of Mettl14 and p38.

(**a**) qPCR analysis of *p38* mRNA level from si-Ctrl and *p38α*-knockdown (si-*p38*) mouse peritoneal macrophages. (**b**, **c**) Immunofluorescence staining for Mettl14 in si-*Ctrl* and si- *p38α* mouse peritoneal macrophages stimulated with PGN for 2 h. Scale bar, 10 μm. Every point in (**b**) represents the number of Mettl14 foci in 30 cells. The foci in the nucleus with a diameter over 200nm were included. **(d)**Analysis of

intensity of Mettl14 foci in mouse peritoneal macrophages stimulated with PGN by Image J**.** Related to Fig. 6a. (**e**) Immunoblot (IB) of p38 in peritoneal macrophages isolated form $p38^{f/f}$ or $p38^{-/-}$ mice. (**f**) ELISA assay verifies the specificity of mouse monoclonal p-Mettl14 antibody. (**g**) In vitro kinase assay of purified recombinant GST-p38 (active) with Mettl14-WT and Mettl14-T72A. (**h**) Immunoblot (IB) and immunoprecipitation (IP) of HEK293T cells transfected with plasmids encoding FLAG-EsxB for 48 h, IP IgG was negative control. (**i**) Immunoblot (IB) and immunoprecipitation (IP) of mouse peritoneal macrophages infected with H37Rv for indicated times (MOI = 5). IP IgG was negative control. (**j**) iBMDM cell was transfected with vector or Flag-EsxA, then infected with H37Rv or H37Rv(ΔEsxB) for indicated times (MOI = 5). Cell was harvested, immunoblot (IB) and immunoprecipitation (IP) test the interaction of p38 and Mettl14. (**k**) Immunoblot (IB) and immunoprecipitation (IP) of mouse peritoneal macrophages infected with H37Rv or H37Rv(ΔEsxB) for indicated times (MOI = 5). (**l**) MeRIP-qPCR analysis of relative m^{6}A level of *Nox2* mRNA in peritoneal macrophages from $p38\alpha^{f/f}$ or $p38\alpha^{-/-}$ mice infected with H37Rv or H37Rv(ΔEsxB) for 2 h. (**m**) qPCR analysis of *Nox2* mRNA in mouse peritoneal macrophages treated with DMSO or p38 inhibitor SB203580 (10 μM) following H37Rv or H37Rv(ΔEsxB) infection for 4 h (MOI = 5). (**n**) Changes in the levels of ROS (DCF staining; green) in mouse peritoneal macrophages treated with DMSO or p38 inhibitor SB203580 (10 μM) following H37Rv or H37Rv (ΔEsxB) infection for 4 h (MOI = 5). ELISA experiment is performed only once in **f**. In vitro kinase assay in **g** is repeated twice. Results in **h-k** is representative images from one of three independent experiments. All of the bar graphs in this figure reflect the mean ± s.e.m from three independent biological experiments. Two-tailed unpaired Student's t-test were used.

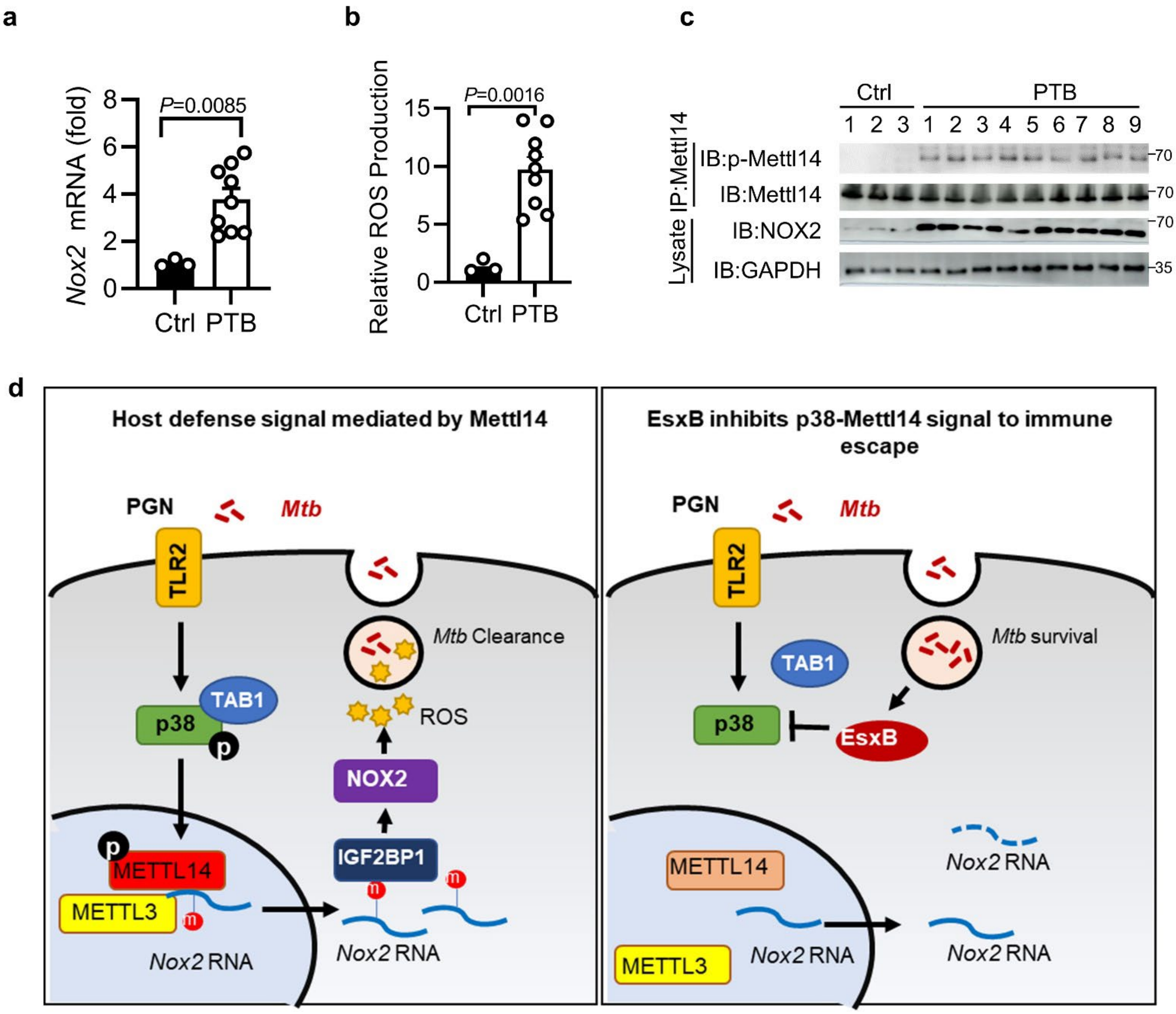

Supplementary Fig.S7 METTL14 phosphorylation clinically associates with Nox2 mRNA level

(**a-c**) qPCR analysis of *Nox2* mRNA(**a**), changes in the levels of ROS (**b**) from BALF cells of TB-negative patients (n = 3) and pulmonary TB patients (n = 9). (**c**) Immunoblot (IB) from BALF cells of TB-negative patients (n = 3) and pulmonary TB patients (n = 9). Two-tailed unpaired Student's t-test were used. (**d**) Summary diagram: in macrophages, PGN activates p38 which directly phosphorylates METTL14 on T72 and triggers its phase separation mediated recruitment of METTL3, thus promotes m^{6}A methylation of *Nox2* RNA. *Nox2* RNA m^{6}A methylation was recognized by m6A binding protein IGF2BP1 which increases the RNA stability to enhance ROS production and *M. tuberculosis* intracellular clearance. However, *M. tuberculosis* secreted protein EsxB (CFP10) interacts with p38 and inhibits the TAB1-mediated p38 autophosphorylation and the interaction of p38 with METTL14, therefore blocks *Nox2* RNA m^{6}A methylation and macrophages ROS production to achieve immune escape.

Supplementary Table S1

Name (Strains/Plasmids /Primers)	Description	Reference
Strain-*E. coli* DH5α	F- φ80lacZ△M15△ (lacZYA-argF) U169 endA1 recA1hsdR17(rk-,mk+) supE44λ-thi-1 gyrA96 relA1 phoA	Tiangen
Strain-*M. tuberculosis*	H37Rv	Gene-optimal
Strain-M. tuberculosis ΔEsxB	H37Rv (ΔEsxB)	Gene-optimal
Strain-M. tuberculosis ΔEsxB+GFP	H37Rv(ΔEsxB+GFP)	This study
Strain-M. tuberculosis ΔEsxB+EsxB	H37Rv(ΔEsxB+EsxB)	This study
Plasmid-pMV261	Mycobacterial extrachromosomal expression vector, Kan^R	K.Mi
Plasmid-pMV261-GFP	For overexpression of GFP in ΔEsxB	J.Yu
Plasmid-pMV261-EsxB	For overexpression of EsxB in ΔEsxB	Gene-optimal
Plasmid-pcDH-FLAG-Mettl14	For expression of FLAG-Mettl14 in mammalian cells	This study
Plasmid-pcDH-HA-Mettl14	For expression of HA-Mettl14 in mammalian cells	This study
Plasmid-pcDH-FLAG-Mettl14 T72A	For expression of FLAG-Mettl14 mutants in mammalian cells	This study
Plasmid-pcDNA-FLAG-TRAF6	For expression of FLAG-TRAF6 in mammalian cells	D.P.Yan
Plasmid-pcDNA-FLAG-P38	For expression of FLAG-P38 in mammalian cells	D.P.Yan
Primer-m-gapdh-f	ACGGCCGCATCTTCTTGTGCA	
Primer-m-gapdh-r	ACGGCCAAATCCGTTCACACC	
Primer-m-Mettl14-f	CAGCAGGAGGAAGAGAAT	
Primer-m-Mettl14-r	TGTTGGCAGTAATCATTATGG	
Primer-nox2-f	CTACCTAAGATAGCAGTTGA	
Primer-nox2-r	CTAACATCACCACCTCATA	
Primer-R-RT-IGF2BP1	TCCTGGATAGTCTAATTCTGAT	
Primer-F-RT-IGF2BP1	CGTGGATCGGTACATACT	

Mettl14 KI donor	ggcttcctatgatacatctgctccaaactcaaaacggaagtgtctggacgaaggagaggctgatgaagacaaagtagaagaatataaggcaagtggaaatgagatagttttacgcatggc	
Primer:Mettl14 RNA-F:	TAATACGACTCACTATAGGGAAACGGAAGTGTCTGGATGAGTTTTAGAGCTAGAA	
Primer:Mettl14 RNA-R:	AAAAAAGCACCGACTCGGTG	
GLuc Forward Primer	CGACATTCCTGAGATTCCTGG	
GLuc reverse Primer	TTGAGCAGGTCAGAACACTG	
cCLuc Forward Primer	GCTTCAACATCACCGTCATTG	
cCLuc Reverse Primer	CACAGAGGCCAGAGATCATTC	
mouse Mettl14: siRNA1	GCAUGGUGUGUUAAAUA	
mouse Mettl14: siRNA2	GAGUUAAUAGCUAAGUCAAAC	
mouse Mettl14: siRNA3	GGAUGAUAUUAUGAAGUUAGA	
mouse Nox2: siRNA1,	GACAAGGAUUCGAAGACAACU	
mouse Nox2: siRNA2,	GUGAAGAUGUGUUCAGCUAUG	
mouse Nox2: siRNA3,	AGUGCGUGUUGCUCGACAAGG	
mouse p38α: siRNA1	GGUCUUGUGUUUAGGUCAAGG	
mouse p38α: siRNA2	ACCUCAGUGUGCAGUUCAACU	
mouse p38α: siRNA3	CACUCAUGGUGACAAGCUAAG	
mouse Igf2bp1: siRNA1	UCUACUUUCCCCGAGAAAGUU	
mouse Igf2bp1: siRNA2	UCAAUCUCUAGACGUUUUCCU	
mouse Igf2bp1: siRNA3	AAUGAUAGCGCCUACAUACUG	
p1-down for SELECT	5phos/CTGGACCTTTGTCCCAcagaggctgagtcgctgcat	
p1-up for SELECT	tagccagtaccgtagtgcgtgGCAACACGAAGGTCTG	
